# Activation of the Jasmonic Acid Pathway by Depletion of the Hydroperoxide Lyase OsHPL3 Reveals Crosstalk between the HPL and AOS Branches of the Oxylipin Pathway in Rice

**DOI:** 10.1371/journal.pone.0050089

**Published:** 2012-11-29

**Authors:** Xiaoqiang Liu, Feng Li, Jiuyou Tang, Weihong Wang, Fengxia Zhang, Guodong Wang, Jinfang Chu, Cunyu Yan, Taoqing Wang, Chengcai Chu, Chuanyou Li

**Affiliations:** State Key Laboratory of Plant Genomics, National Centre for Plant Gene Research, Institute of Genetics and Developmental Biology, Chinese Academy of Sciences, Beijing, People's Republic of China; Ghent University, Belgium

## Abstract

The allene oxide synthase (AOS) and hydroperoxide lyase (HPL) branches of the oxylipin pathway, which underlie the production of jasmonates and aldehydes, respectively, function in plant responses to a range of stresses. Regulatory crosstalk has been proposed to exist between these two signaling branches; however, there is no direct evidence of this. Here, we identified and characterized a jasmonic acid (JA) overproduction mutant, *cea62*, by screening a rice T-DNA insertion mutant library for lineages that constitutively express the AOS gene. Map-based cloning was used to identify the underlying gene as hydroperoxide lyase *OsHPL3*. *HPL3* expression and the enzyme activity of its product, (*E*)-2-hexenal, were depleted in the *cea62* mutant, which resulted in the dramatic overproduction of JA, the activation of JA signaling, and the emergence of the lesion mimic phenotype. A time-course analysis of lesion formation and of the induction of defense responsive genes in the *cea62* mutant revealed that the activation of JA biosynthesis and signaling in *cea62* was regulated in a developmental manner, as was OsHPL3 activity in the wild-type plant. Microarray analysis showed that the JA-governed defense response was greatly activated in *cea62* and this plant exhibited enhanced resistance to the T1 strain of the bacterial blight pathogen *Xanthomonasoryzaepvoryzae* (*Xoo*). The wounding response was attenuated in *cea62* plants during the early stages of development, but partially recovered when JA levels were elevated during the later stages. In contrast, the wounding response was not altered during the different developmental stages of wild-type plants. These findings suggest that these two branches of the oxylipin pathway exhibit crosstalk with regards to biosynthesis and signaling and cooperate with each other to function in diverse stress responses.

## Introduction

Plants have evolved complex signaling pathways to coordinate effective responses to biotic and abiotic challenges, and to developmental stimuli. The oxylipin pathway, which induces the synthesis of biologically active metabolites called oxylipins upon activation by various stimuli, is a lipid-based signaling network. The pathway is composed of several competing branch pathways that play pivotal roles in an array of biological functions, including reproduction, in responses to biotic and abiotic stresses, in metabolic processes and in developmental regulation. Oxylipin biosynthesis is initiated by the coordinated action of lipases and lipoxygenases, which generate highly active hydroperoxy fatty acids, including 9-/13-hydroperoxy-octadecadienoic acids (9-/13-HPODs) and 9-/13-hydroperoxy-octadecatrienoic acids (9-/13-HPOTs) [1]. These hydroperoxides are substrates for a group of enzymes present in four different branches of the pathway, namely, peroxygenase (POX), divinyl ether synthase (DES), allene oxide synthase (AOS) and hydroperoxide lyase (HPL) [1], [2]. Among these, the AOS and HPL branches, which are responsible for the production of jasmonates and aldehydes, respectively, are the best studied and are known to be involved in multiple developmental and defense pathways, especially those involved in herbivore resistance, disease resistance and general stress responses [1], [3].

A large body of genetic and molecular evidence shows that jasmonates play a central role in plant defense responses to pathogen infection and insect herbivory [3], [4]. Genetic evidence for the role of jasmonic acid (JA) in defense signaling mostly comes from research on *Arabidopsis thaliana* mutants impaired in JA biosynthesis or perception, which lack effective defense responses against pathogen infection [5]–[9]. In addition, Arabidopsis mutants with constitutive activation of the JA signaling pathway (e.g., *cev1*) display enhanced resistance to bacterial and fungal pathogens [10], [11]. Furthermore, jasmonates have also been shown to play important roles in defense responses in *Solanum lycopersicum* (tomato), *Nicotiana tabacum* (tobacco) and *Solanum tuberosum* (potato) plants through mutant or transgenic plant analysis [12]–[14]. In monocotyledonous plants, most of our information comes from studies of the effect of exogenous JA or methyl jasmonate (MeJA) treatments on the induction of defense genes, such as pathogenesis-related (PR) genes, including *PR1b*, *PR2*, *PR5* and *PR10* [15]–[17], and phytoalexins, such as sakuranetin and momilactone A in *Oryza sativa* (rice) leaves [18], [19]. Molecular, biochemical and pathological analyses of transgenic rice lines in which oxylipin biosynthesis genes (e.g., *OsAOS2*, *OsOPR1* and *OsHPL2*) or transcription factor genes were overexpressed, revealed that JA plays a significant role in defense gene activation, and host resistance to the rice blast fungus and bacterial blight pathogen *Xanthomonasoryzaepvoryzae* (*Xoo*) [20]–[24]. However, the role of JA signaling in defense responses in rice is still not clear. Therefore, the role of JA in the rice defense response needs to be further elucidated through robust genetic and molecular analyses using rice mutants or transgenic lines with altered JA biosynthesis or signaling pathways.

Whereas much research has focused on the functions of the jasmonates generated by the AOS branch pathway, the HPL branch pathway, which has recently been implicated in plant defense processes, is not fully characterized. Several studies have suggested that crosstalk exists between these two competing branches of the oxylipin pathway. For instance, the metabolites of the HPL branch of the pathway activate defense signaling, eliciting the accumulation of phytoalexins in *Gossypium hirsutum* L. (cotton) [25], producing anthocyanins in Arabidopsis [26], and resulting in the accumulation of the systemin precursor in *Solanum lycopersicum* (tomato) [27]. C_6_ volatiles up-regulated the expression of some defense genes involved in phenylpropanoid and oxylipin synthesis in Arabidopsis [28] and *Citrus jambhili* [29]. HPL genes are induced by mechanical wounding [30], [31], and expression varies greatly with developmental stage and tissue type [32], [33]. Because the accumulation of induced transcripts is not correlated with instant responses in downstream products, posttranscriptional control mechanisms, involving substrate availability or compartmentation, are expected to be involved in the regulation of HPL expression [32], [34]. The HPL gene family can be classified into two groups, 13-HPLs and 9-/13-HPLs, based on the substrate specificities. HPL acts on 13-HPOT/HPOD to yield the C_6_ aldehydes, i.e., hexenals and hexanals, which have been suggested to play signaling roles in plant defense processes [26], especially those involved in herbivore resistance [35], disease resistance [36] and plant-insect communication [37]. The functional roles of the HPL branch of the pathway have also been genetically manipulated in transgenic plants in which HPL activity was silenced [38], [39]. For example, antisense-mediated HPL depletion in transgenic potato plants resulted in impaired sucking insect aphid resistance, implying that the HPL-catalyzed production of C_6_ aldehydes may be a key step in a built-in resistance mechanism of plants against some sucking insect pests [38]. Furthermore, *OsHPL2*-overexpressing transgenic rice, in which (*E*)-2-hexenal accumulated abundantly, exhibited enhanced resistance to bacterial blight [23]. There was a negative correlation between the products of the AOS and HPL branches of the oxylipin pathway. An overlap of GLV- and JA-regulated transcript accumulation in the transformed antisense-NaHPL (as-hpl) and antisense-NaAOS (as-aos) plants revealed that the two signaling cascades had different roles in defense responses and suggested that crosstalk exists between these two branches of the oxylipin pathway with respect to biosynthesis and signaling [39]. However, the mechanism that regulates this crosstalk remains unknown.

Here, we identified a JA overproduction mutant, *cea62*, in a genetic screen for lineages with constitutively active expression of an allene oxide synthase gene in a rice T-DNA insertion mutant library. We then used a map-based cloning strategy to identify the target gene as *OsHPL3*. The *cea62* mutant constitutively expressed *OsAOS* and exhibited spontaneous disease-like lesions and enhanced resistance to bacterial blight compared with the wild-type plant. Furthermore, we revealed that the mutant phenotype was correlated with increased jasmonic acid (JA) accumulation, induction of JA biosynthesis genes and activation of the JA signaling pathway. The microarray data showed that a series of defense response genes was also elevated in the mutant plant. In contrast, the HPL-derived oxylipin (*E*)-2-hexenal was dramatically reduced in the mutant. These results demonstrate that the OsHPL3-derived oxylipin pathway also plays an important role in normal plant growth and development, and that these processes are dramatically affected when the function of *OsHPL3* is abolished. Furthermore, we investigated whether crosstalk exists between the HPL and AOS oxylipin biosynthesis and signaling pathways, especially during defense responses.

## Results

### Characterization of the *cea62* mutant

**Figure 1:**
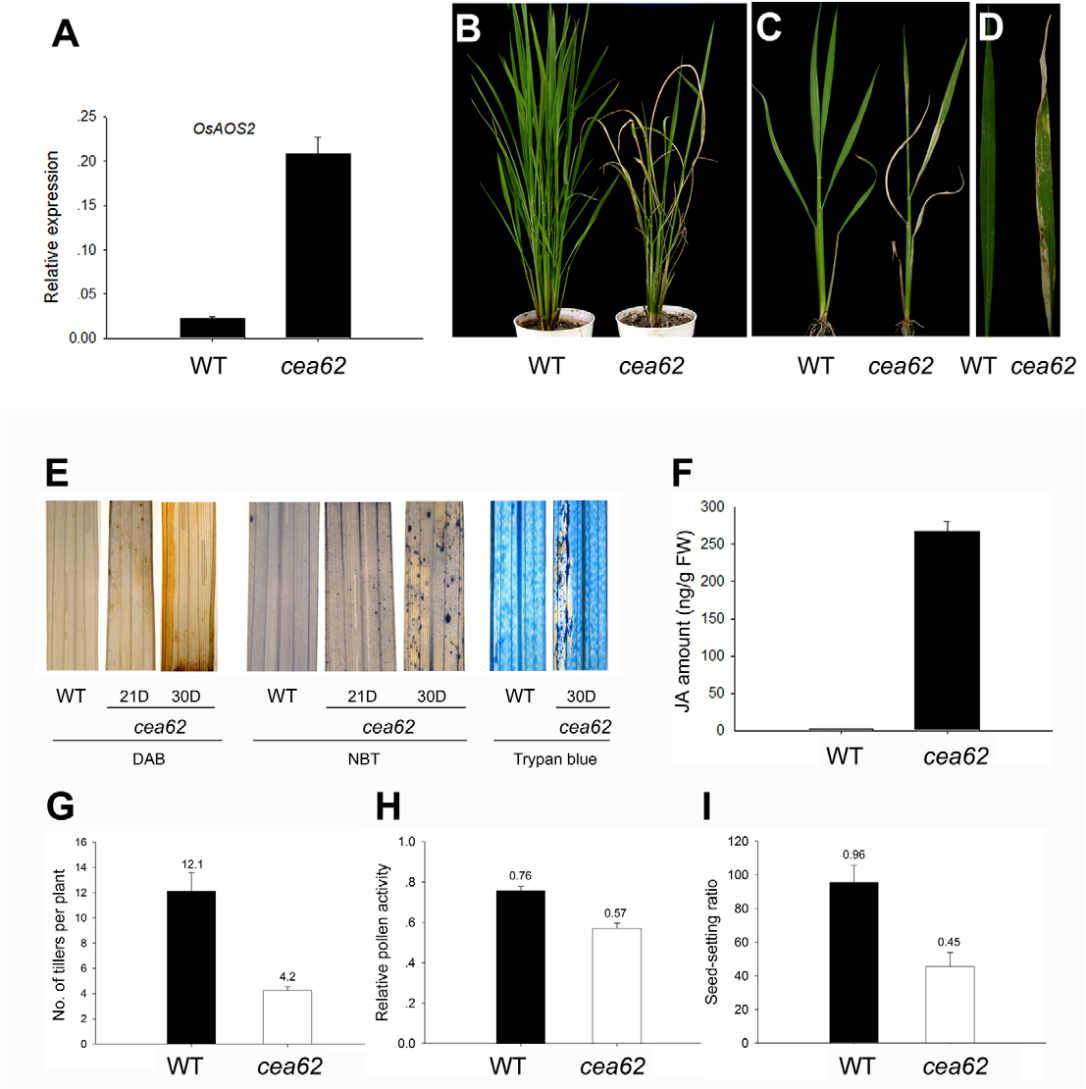
Phenotype of the *cea62* mutant. *OsAOS2* expression was strongly induced in the *cea62* mutant (A). Two-month-old plants (B), tillers (C) and leaves (D) of the wild type (WT) and *cea62* mutant. (E) The expression of several histochemical markers in *cea62* and wild-type plants was analyzed. Leaves were stained with DAB, NBT or trypan blue at 21 or 30 days after sowing. And JA production (F) was much greater in the *cea62* mutant than in the wild type. (G–I) Quantification of several agricultural traits, including tiller number (G), seed setting ratio (H), and relative pollen activity (I). Each bar is the mean ± SD of 30 replicate samples.

To gain insight into the molecular basis of the JA signaling pathway in rice, we identified a novel JA overproduction mutant, *cea62*, by monitoring the expression of the Constitutive Expression of Allene oxide synthase gene (*cea*) in our rice T-DNA insertion mutant population (*Oryza sativa* var. Nipponbare background) [40]. The expression of *OsAOS2* was greatly elevated in the *cea62* mutant compared to in the wild-type plant (Fig. 1A), and the mutant showed a lesion mimic (LM) phenotype in the absence of pathogen attack (Fig. 1B, C and D). Brown lesion spots appeared on leaves about 2 weeks after sowing and gradually spread over the entire leaf surface during the subsequent developmental stages. To identify the biochemical mechanisms underlying the development of hypersensitive response (HR)-like lesions in the *cea62* mutant, we examined the expression of several histochemical markers in both the *cea62* mutant and wild-type plants. DAB staining, which is indicative of H_2_O_2_ accumulation, was strictly correlated with lesion formation in the *cea62* mutant, whereas wild-type leaves produced no H_2_O_2_ (Fig. 1E). Similarly, purple formazan precipitates formed on the *cea62* mutant stained with NBT (nitro-blue tetrazolium chloride), an indicator of O_2_^−^, but not on any of the wild-type leaves. Furthermore, cells at the site of lesions in the *cea62* mutant stained deep blue upon trypan blue staining, which indicates irreversible membrane damage or cell death, whereas the leaves of wild-type plants did not stain with trypan blue (Fig. 1E). These data suggest that reactive oxygen species (ROS) in the mutant leaves induced the HR-like cell death and are responsible for the lesion mimic phenotype. JA production was increased hundreds of times (Fig. 1F). Furthermore, the *cea62* plants have reduced stature, a decreased tiller number and a decreased seed setting ratio, which is mainly due to decreased pollen fertility compared with that of the wild-type plant (Fig. 1G–I, and Fig. S1A, B).

### Map-based cloning of *CEA62* and its expression patterns

**Figure 2:**
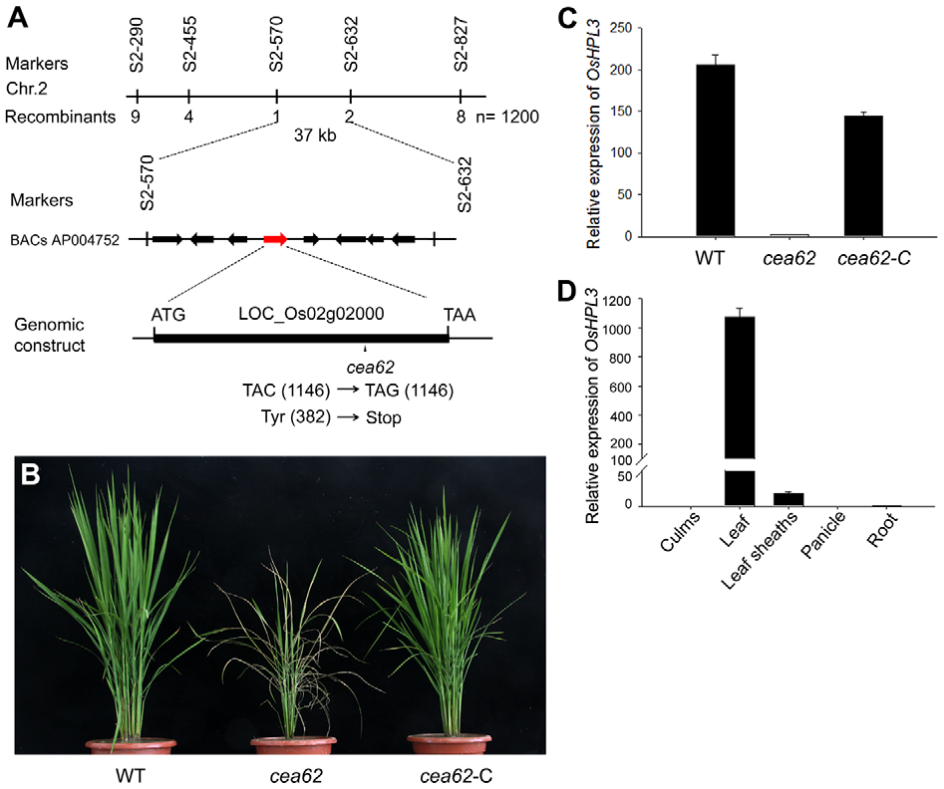
Molecular identification of the *cea62* mutant. (A) Map-based cloning of *cea62*. The *CEA62* gene (red arrow) was mapped to the short arm of chromosome 2 between markers S2-570 and S2-630 and was delimited to a 37-kb region with eight candidate genes. A single-base substitution (TAC→TGA) at the 1146^th^ position of LOC_Os02g02000 was present in the *cea62* mutant. (B) Functional complementation of the *cea62* mutant. Three-month-old wild type, *cea62* and complemented *cea62* (*cea62-*C) plants are shown. (C) Expression of *OsHPL3*/*CEA62* in the wild type (WT), *cea62* mutant and *cea62-*C plants and (D) expression of *OsHPL3*/*CEA62* in different tissues of the unwounded wild type, as analyzed by quantitative RT-PCR. Rice *OsACT* was used as an internal control. These data were obtained from three independent replicates. Each bar is the mean ± SD of three replicate samples.

Genetic analysis revealed that *cea62* was caused by a single recessive nuclear gene mutation that did not co-segregate with the T-DNA insertion (data not shown). Therefore, we used map-based cloning to isolate the underlying gene. An F2 mapping population was constructed by crossing *cea62* (Japonica) and Minghui 63 (Indica). Using 1200 F2 plants, the candidate gene was narrowed down to a 37-kb region between markers S2-570 and S2-632 on chromosome 2 (Fig. 2A). There are eight predicted genes within this region (Fig. 2A and Table S1). After sequencing these genes, a single-base substitution (TAC→TAG) was identified at the 1146^th^ position of LOC_Os02g02000 in *cea62*, which generates a premature stop codon (Fig. 2A and Fig. S2A). To verify that Os02g02000 is *CEA62*, we performed a genetic complementation test in which *cea62* plants were transformed with wild-type Os02g02000, which consists of 6930 bp of genomic DNA containing the entire coding region driven by its native promoter (Data S1). The resulting complemented plants are referred to hereafter as *cea62*-C. The transgene complemented the *cea62* phenotype (Fig. 2B), confirming that Os02g02000 is indeed *CEA62* and that this single-base substitution is responsible for the phenotype of *cea62* plants. *CEA62* encodes a cytochrome P450 family protein, which is a hydroperoxide lyase, OsHPL3 [41]. OsHPL3 is predicted to encode a polypeptide of 488 amino acids and is classified as a 13-HPL [30], [31], as it uses 13-hydroperoxides as substrates [41]. Three HPLs are present in the rice genome, OsHPL1, OsHPL2 and OsHPL3. OsHPL3 belongs to the CYP74B subfamily and shares less sequence similarity with OsHPL1 and OsHPL2, which belong to the CYP74C subfamily (Fig. S2B). Real-time PCR analysis showed that *OsHPL3* transcripts were totally abolished in the *cea62* mutant and were almost at wild-type levels in the complemented *cea62* transgenic plant (*cea62*-C) (Fig. 2C). We propose that a mechanism of non-sense mediated decay (NMD) plays a role in this abnormal mRNA clearance. The organ-specific expression pattern of *OsHPL3* was further examined. *OsHPL3* was expressed in leaf blades and weakly in leaf sheaths, but was not expressed in culms, panicles and roots (Fig. 2D). These data confirm that *OsHPL3* is *CEA62* and that depletion of *OsHPL3* caused the phenotype of the *cea62* mutant.

### Mutation of *OsHPL3* causes a reduction in C_6_ Aldehyde and JA accumulation in *cea62* plants

**Figure 3:**
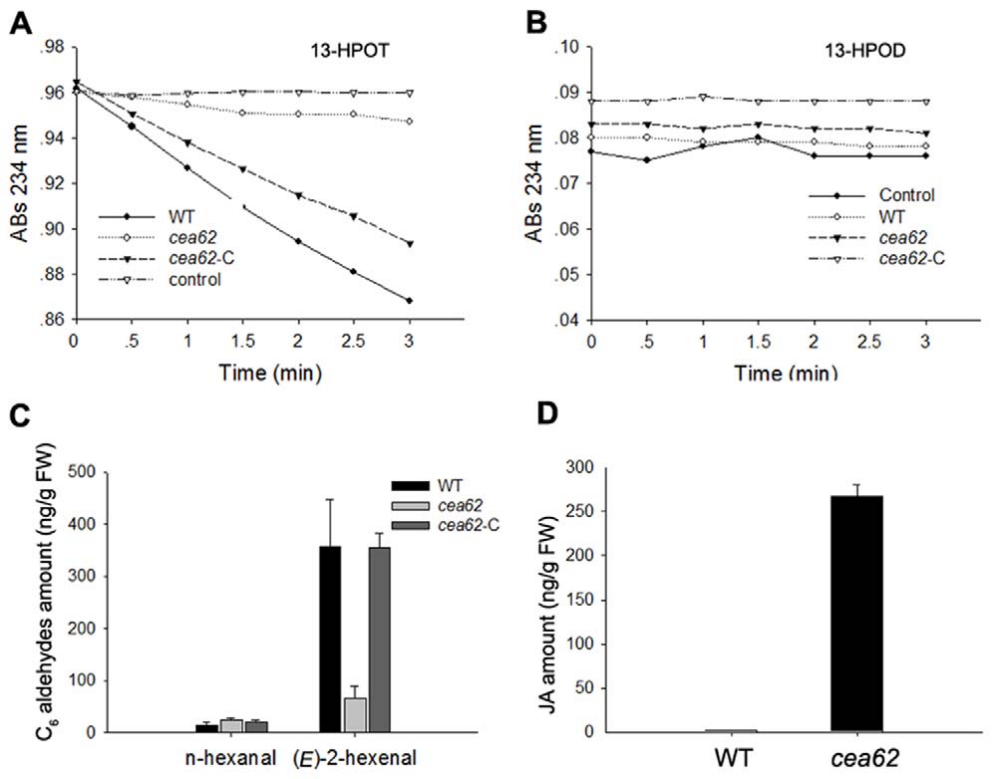
In vitro enzyme activity assays of OsHPL3 and quantification of C_6_-aldehydes and JA. (A) OsHPL3 activity was monitored by the loss of absorbance of substrate at 234 nm in leaf extracts of the wild type (WT), *cea62* mutant and *cea62* complemented transgenic plant (*cea62*-C) using 13-hydroperoxylinolenic acid (13-HPOT) or (B) 13-hydroperoxylinoleic acid (13-HPOD) as substrate. Results were obtained from one experiment with three biological replicates, and similar results were obtained in three independent experiments. (C) The levels of n-hexanal and *(E)-*2-hexenal in the WT, *cea62* and *cea62*-C. (D) The quantity of JA in the WT and *cea62* mutant. Histograms are the average of triplicate assays and the bars indicate SD.

*OsHPL3* transcripts were almost abolished in the *cea62* mutant. Consequently, we propose that the ability of OsHPL3 to metabolize the cleavage of 13-hydroperoxides is greatly impaired in *cea62* plants. We detected OsHPL3 enzyme activity using 13-HPOT as a substrate in the *cea62* mutant, and found that enzyme lysate of *cea62* plants largely failed to degrade 13-HPOT, whereas 13-HPOT was metabolized quite well by enzyme lysate extracted from the wild-type and complemented lines (Fig. 3A). This result indicates that mutation of the *OsHPL3* gene abolished OsHPL3 enzyme function in the *cea62* mutant. We also used 13-HPOD as a substrate to perform the same enzyme assay. No enzyme activity was detected for the wild type, *cea62* mutant and *cea62*-C when 13-HPOD was used as substrate (Fig. 3B).

We next quantified the HPL pathway metabolites, i.e., the C_6_ aldehydes hexanal and hexenal, in the *cea62* mutant, wild type and *cea62*-C. The *OsHPL3* mutation resulted in a significant reduction in the level of (*E*)-2-hexenal in the *cea62* mutant compared with in wild-type and *cea62*-C plants (Fig. 3C). In contrast, there was no difference in the level of the product of 13-HPOD, hexanal, between the wild-type, *cea62* mutant and *cea62*-C plants (Fig. 3C). This indicates that the mutation in *OsHPL3* abolishes the ability of the encoded protein to catalyze the cleavage of 13-HPOT into (*E*)-2-hexenal. As the HPL pathway competes against the AOS pathway for the same substrate, 13-HPOT, it is presumed that blocking of the HPL branch would have some effect on the AOS branch pathway. However, the level of JA was 162 times greater in the *cea62* mutant than in the wild-type plant (Fig. 1F). This suggests that the mechanism underlying substrate competition might not be the only cause of this significant induction of JA. These data showed that mutation of OsHPL3 not only impaired the production of its metabolites, but also induced the overproduction of JA. Furthermore, these findings suggest that crosstalk exists between HPL and AOS oxylipin biosynthesis and/or signaling pathways.

### Depletion of OsHPL3 activates the JA signaling pathway in a development-dependent manner that resembles that of OsHPL3 activity in the wild type

**Figure 4:**
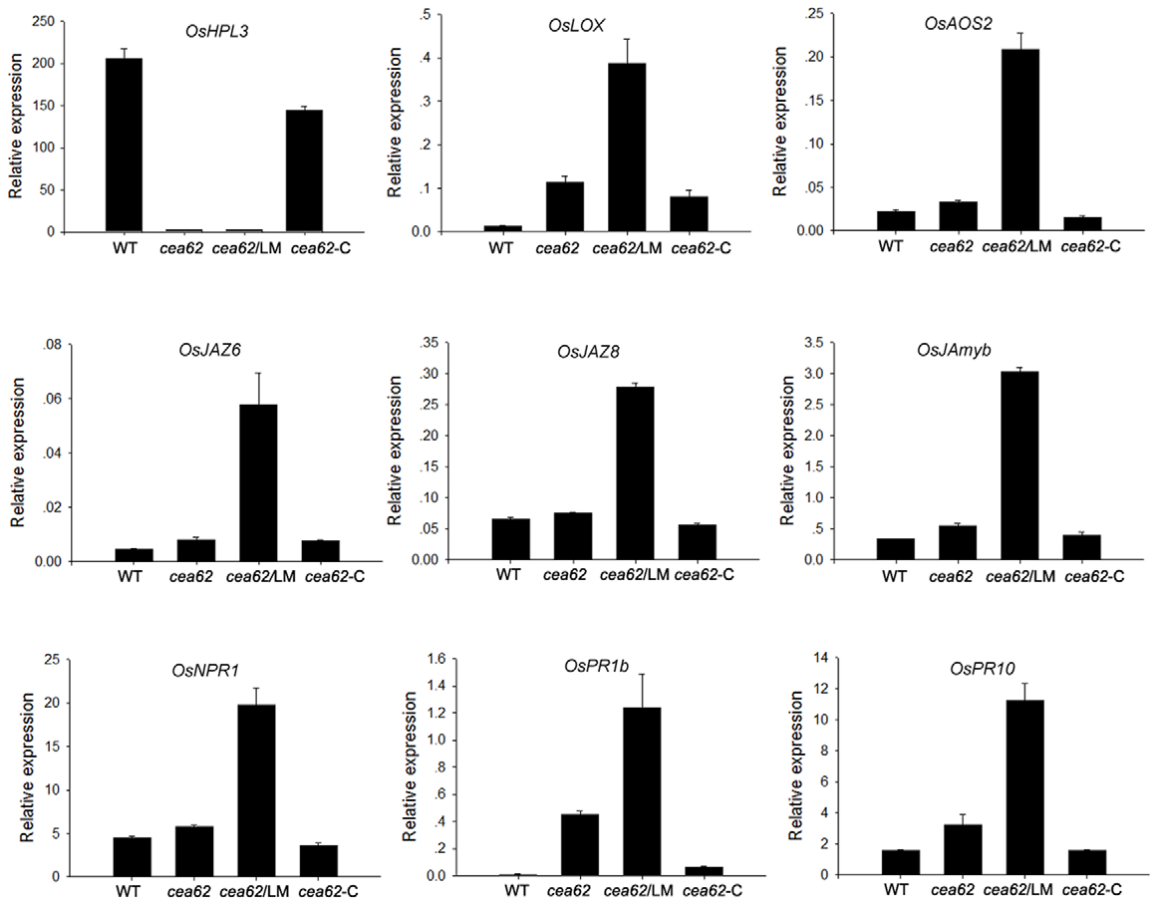
Expression patterns of JA-related genes in the wild type and *cea62* mutant. *OsLOX* and *OsAOS2* are JA biosynthesis genes in rice. *OsJAZ6*, *OsJAZ8* and *OsJAmyb* are JA signaling genes in rice. *OsPR1b* and *OsPR10* are pathogen-related genes in rice. *OsNPR1* is a key regulator of an SA-dependent systemic resistance gene. Rice *Actin1* was used as a reference control. Leaves of the wild type (WT), the *cea62* mutant at two weeks (*cea62*) and three months (*cea62*/LM) after sowing and the *cea62* complemented transgenic (*cea62*-C) plant were used to monitor the expression of these marker genes. In all panels, the mean is based on the average of three biological repeats. Each bar is the mean ± SD of three replicate samples.

**Figure 5:**
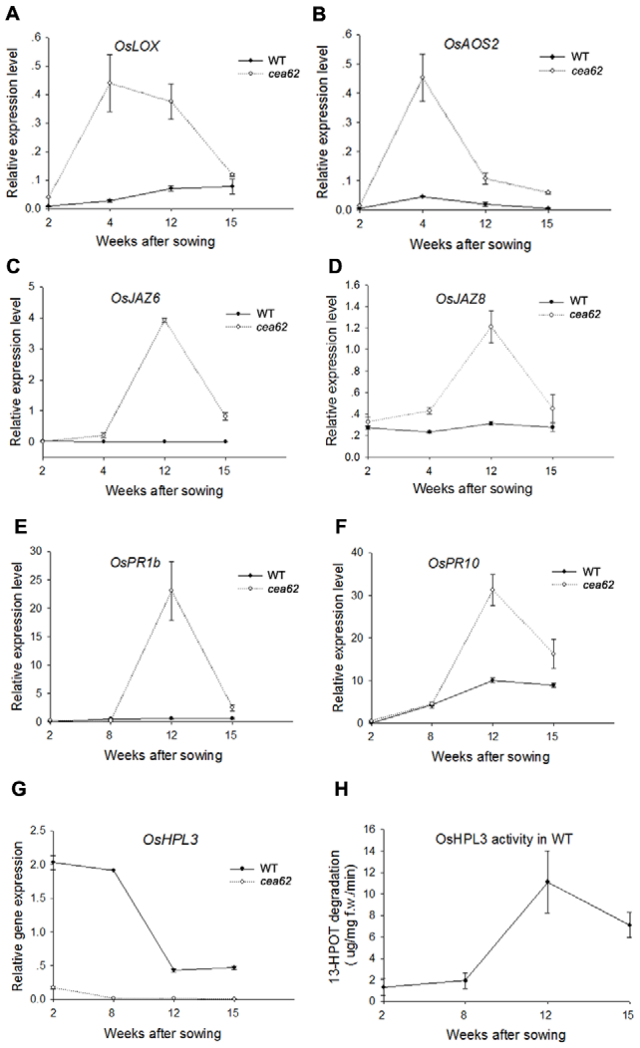
Expression patterns of *OsHPL3* and JA-related maker genes in the wild type and *cea62* mutant at different developmental stages. The expression of JA biosynthesis genes, (A) *OsLOX* and (B) *OsAOS2*; JA signaling genes, (C) *OsJAZ6* and (D) *OsJAZ8*; defense-related maker genes, (E) *OsPR1b* and (F) *OsPR10*; and (G) *OsHPL3* were monitored at various time points. The enzyme activity of (H) OsHPL3 was also recorded. These data were obtained from three independent replicates. Each bar is the mean ± SD of three replicate samples.

We next examined whether the JA signaling pathway was ubiquitously activated in the *cea62* mutant plant by monitoring the expression of JA signaling pathway genes. We found that the expression of a series of JA biosynthesis and signaling pathway genes, such as *OsLOX*, *OsAOS2*, *OsJAZ6*, *OsJAZ8* and *OsJAmyb*, was greatly induced in *cea62* plants after the LM phenotype appeared (i.e., three months after sowing). However, the expression of these JA-related genes in *cea62* plants was only slightly upregulated or the same as that in wild-type and *cea62*-C plants before the LM phenotype appeared, two weeks after sowing (Fig. 4). Similarly, the expression of some disease-related marker genes, such as *OsNPR1*, *OsPR1b* and *OsPR10*, in *cea62* plants exhibited the same pattern as that of JA-related genes (Fig. 4). We further monitored the expression of these genes at different developmental stages in wild-type and *cea62* plants. The expression of these JA-related genes was maintained at or a little above basal levels during all of these developmental stages in the wild-type plant, whereas in the *cea62* mutant plant, the expression of JA biosynthesis genes, such as *OsLOX* and *OsAOS2*, began to dramatically increase two weeks after sowing (WAS) and peaked at four weeks after sowing, before gradually declining to basal levels by fifteen weeks after sowing (Fig. 5A and B). For *OsJAZ6* and *OsJAZ8*, there was a two-week delay in induction compared with that of JA biosynthesis genes in the *cea62* plant, and the expression of these genes peaked twelve weeks after sowing (Fig. 5C and D). Furthermore, the expression of *OsPR1b* and *OsPR10* started to increase eight weeks after sowing and peaked at twelve weeks after sowing in *cea62* plants (Fig. 5E and F). These data suggest that the activation of JA biosynthesis, signaling pathway and defense response by depletion of OsHPL3 varied at different developmental stages, and was associated with the development of the lesion mimic phenotype.

Previous studies showed that transgenic plants in which the HPL gene was knocked-down by RNAi or anti-sense technology did not display any developmental phenotypes [38], [39]. Furthermore, it was even concluded that HPL activity was dispensable for normal growth and development in the Arabidopsis Columbia (Col) ecotype [42]. Here, we identified a natural *HPL* mutant, *cea62*, which displayed a severe phenotype, i.e., lesions on the leaves and premature leaf senescence, in rice (Fig. 1A). This indicated that OsHPL3 plays an important role in normal rice growth and development.

To gain insight into OsHPL3 function in normal growth and development, we monitored the expression of *OsHPL3* and the enzyme activity of its encoded protein at 2, 8, 12 and 15 weeks after sowing in wild-type plants. The *OsHPL3* transcript level was elevated up to 8 weeks after sowing and was greatly reduced after 12 weeks in wild-type plants, but was absent in *cea62* plants at all developmental stages (Fig. 5G). In contrast, OsHPL3 activity was low before 8 weeks after sowing and increased greatly by 12 weeks after sowing (Fig. 5H). This result suggests that *OsHPL3* expression is regulated by developmental cues at both the transcriptional and protein levels, which may involve changes in subcellular localization and protein modifications [32]. All these data demonstrate that a reduction in OsHPL3 activated JA biosynthesis, signaling and defense responses, and that this activation varied at different developmental stages in the *cea62* mutant, as did the activity of OsHPL3 in the wild type. Furthermore, these data indicate that the relationship between the HPL and AOS branches of the oxylipin pathways is much more intricate than simple substrate competition, and that crosstalk exists between the transcriptional and developmental regulators of these two branches in rice.

### OsHPL3 depletion activates the expression of JA-regulated defense genes in *cea62* plants

To examine gene expression in the *cea62* mutant, we performed a cDNA microarray analysis of rice. We sought to identify genes that were differentially expressed in wild-type and *cea62* plants and thus potentially arose from depletion of the *OsHPL3* transcript and HPL activity. Using the Affymetrix Rice Genome Array, which contains a total of 51,279 transcripts, including 48,564 japonica transcripts and 1,260 indica transcripts, we compared the gene expression profiles in leaf tissues of the *cea62* mutant and wild-type plant after the lesion mimic phenotype appeared two months after sowing (Fig. S3). Table S2 shows that 1686 expressed transcripts (2-fold cutoff, p-values<0.05) were detected. Of these transcripts, 1352 were up-regulated and 334 were down-regulated in the *cea62* mutant compared with those in the wild-type plant (Table S2). Functional classification of the differentially expressed genes revealed that the most significant difference in the gene expression profiles was that the defense responsive genes were greatly activated. These genes included a batch of pathogenesis and disease resistance-related genes, such as *PAL*, *GST*, *PRs*, *NPR1*, and dozens of WRKY transcription factor genes, together with a series of genes involved in JA biosynthesis and signaling pathways (Table 1). We also noticed that ethylene and gibberellin response genes, such as *GA20ox*, *GID1*, *ERF2*, *ERF3*, and ACC oxidase gene, were significantly induced in the mutant (Table 1). Interestingly, some abiotic responsive genes, such as salt- and drought-induced and ABA responsive genes, were greatly induced in *cea62* (Table 1). We further verified the microarray data by real-time quantitative PCR of these genes (Fig. S4). These data suggest that JA-regulated defense responses are significantly activated in the *cea62* mutant.

**Table 1:** Selected differentially expressed genes functionally classified in the *cea62* mutant compared with the wild type by microarray analysis.

Accession num.	fold change (*cea62*/WT)	description and functional categories	qPCR verification (fold change)
**heat shock protein**			
AK063629	152.9	heat shock protein 82	53.1
AF332981	48.1	heat shock protein 101	28.7
AF140500	28.5	heat shock protein 41	41.3
AK071240	13.0	class II small heat shock protein	
AF332981	0.5	heat shock protein 101	
AK287481	0.5	heat shock cognate 70 kDa protein	
**P450 family protein**			
AK119557	386.6	cytochrome P450 72A1	14.9
CT831749	149.1	cytochrome P450 94A1	
AK066760	5.7	cytochrome P450 81E1	
AK071599	5.4	cytochrome P450 71A1	
AK067007	2.2	cytochrome P450 90D2	
**gibberellin related**			
LOC_Os01g61610.1	244.1	gibberellin 20 oxidase 2	207.8
AK107136	46.5	gibberellin receptor GID1L2	39.3
AK101713	16.1	gibberellin 2-beta-dioxygenase	
AB192574	5.3	gibberellin stimulated transcript related protein 1.	7.3
**glutathione S-transferase**			
AF309377	336.9	glutathione S-transferase	349.4
AK107435	330.2	glutathione S-transferase GSTU6	29.3
AF309379	14.6	glutathione S-transferase OsGSTU3	14.5
AF402799	7.4	glutathione S-transferase OsGSTU12	
**Transcription factors**			
LOC_Os11g02540.1	58.1	OsWRKY50 transcription factor	68.6
LOC_Os06g44010.1	36.8	OsWRKY28 transcription factor	28.6
LOC_Os01g60640.1	11.2	OsWRKY21 transcription factor	
AK067834	7.9	OsWRKY62 transcription factor	
LOC_Os12g02440.1	7.8	OsWRKY46 transcription factor	
AK108860	5.8	OsWRKY72 transcription factor	
AK059966	4.9	OsWRKY76 transcription factor	
LOC_Os02g26430.1	4.0	OsWRKY42 transcription factor	
LOC_Os01g51690.1	3.7	OsWRKY26 transcription factor	
AY341856	3.5	OsWRKY16 transcription factor	
AB190436	3.1	OsWRKY53 transcription factor	2.8
AB190817	2.6	OsWRKY71 transcription factor	2.3
AY341849	2.2	OsWRKY8 transcription factor	
CT837881	2.0	OsWRKY7 transcription factor	
AY026332	18.6	Myb transcription factor JAMyb	
CT837881	0.5	OsWRKY7	
AK241326	0.4	WRKY64	
**ethylene responsive factor**			
AK073812	4.5	BTH-induced ERF transcriptional factor 3 (BIERF3)	5.1
CT832402	46.8	ethylene-responsive factor-like protein 1	1.3
LOC_Os07g22730.1	14.8	ethylene-responsive transcription factor 15	899.7
LOC_Os01g54890.1	2.2	ethylene-responsive transcription factor 2	2.0
AK067970	17.6	1-aminocyclopropane-1-carboxylate oxidase	500.0
AK072462	0.3	ethylene-insensitive3-like protein	
**JA synthesis and signaling proteins**			
AK070649	72.9	ZIM motif family protein *OsJAZ6*	329.2
LOC_Os10g25230.1	29.8	ZIM motif family protein *OsJAZ13*	5.5
AK240828	18.5	ZIM motif family protein	
LOC_Os03g08330.1	9.7	ZIM motif family protein *OsJAZ3*	19.9
LOC_Os02g48770.1	739.7	jasmonate O-methyltransferase	
AK241988	203.2	jasmonate O-methyltransferase	
AK066682	168.9	(JAC1) jasmonate-induced protein	
AK100029	3.8	jasmonate O-methyltransferase	2.6
LOC_Os12g37320.1	105.3	lipoxygenase 2.2	
AK073529	67.7	lipoxygenase 2	
AK066737	16.3	lipoxygenase (LOX2)	
AK071915	0.5	lipoxygenase 2.3	
AK105590	26.0	12-oxophytodienoate reductase 2	
AB040743	24.7	12-oxophytodienoic acid reductase (opda2)	
LOC_Os06g11200.1	5.6	12-oxophytodienoate reductase 2	
AK067907	13.9	fatty acid elongase, 3-ketoacyl-CoA synthase,	2.2
CT832317(OSJNBA0054H04.12)	2.3	allene oxide cyclase 4	
AK120087	0.5	ZIM motif family protein	
**senescence related protein**			
AK111790	14.4	senescence-associated protein DIN1,	2.1
AY850134	2.6	senescence-inducible chloroplast stay-green protein	132.1
LOC_Os08g16050.1	2.2	senescence-associated protein DH	
**pathogenesis and disease resistance related protein**			
LOC_Os02g41670.1	22.4	phenylalanine ammonia-lyase	1.1
AK242604	19.0	CSLA11 - cellulose synthase-like family A	
AK070986	6.4	pathogen induced protein 2–4	5.2
LOC_Os12g38170.1	5.1	pathogenesis-related protein 5	9.1
AF395880	3.4	pathogenesis-related protein 1	81.3
LOC_Os07g03600.1	2.1	pathogenesis-related protein PRB1-2	6.6
LOC_Os03g13740.1	4.1	immediate-early fungal elicitor protein CMPG1	
AK240984	3.7	disease resistance RPP13-like protein 1	24.8
AK067801	3.2	phenylalanine ammonia-lyase	
LOC_Os01g50100.1	2.8	multidrug resistance protein 4	
LOC_Os12g07580.1	2.4	disease resistance response protein 206	4.8
LOC_Os11g12040.1	0.4	disease resistance protein RPM1	
AK065363	4.0	regulatory protein NPR1	1.5
**abiotic stress**			
AF001395	122.4	salt-induced protein	2.1
AK100580	6.4	light-inducible protein CPRF-2	209.1
AY056038	2.7	drought-induced S-like ribonuclease	
AK070901	15.2	ABA-responsive protein, GRAM domain protein	9.6
AF300971	2.7	DRE-binding protein 2	1.8

**Fold change between cea62 and WT apices.** (P value represents the Change P value).

### *The cea62* mutant has enhanced resistance to rice bacterial blight (*Xoo*) infection

**Figure 6:**
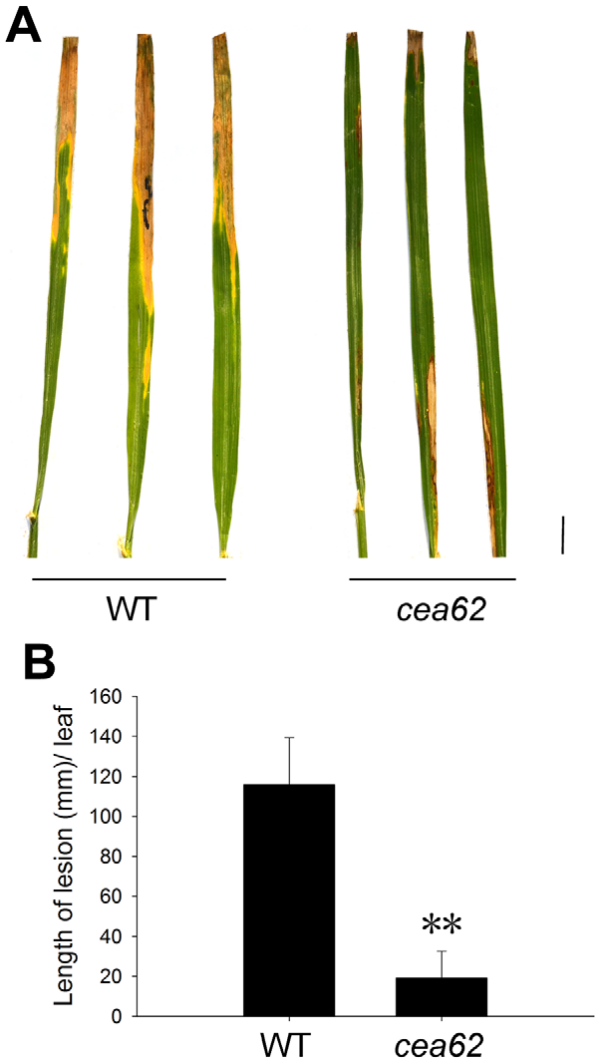
Disease resistance test to rice bacterial blight *Xanthomonasoryzaepvoryzae* (*Xoo*) T1 strains. Photographs were taken at 20 days after inoculation. (A) Lesion lengths on leaves of wild-type (WT) and *cea62* plants at 20 days after inoculation. (B) Data represented here were obtained in one experiment with 30 biological replicates, and similar results were obtained in three independent experiments. Asterisks indicate significant differences in the *cea62* mutant compared with WT plants. (*P<0.05; **P<0.01; Student's *t* test). Scale bars, 1 cm.

To test the disease resistance of *cea62* plants, we infected wild-type leaves and the leaves of *cea62* plants that lacked lesion mimic spots with bacterial blight pathogen *Xanthomonasoryzaepvoryzae* (*Xoo*) T1 strain at about two months after sowing. The *cea62* plants were highly resistant to the T1 strain, whereas the wild-type plants were not. Twenty days after inoculation, the average lesion length on the leaves of the *cea62* mutant was 1.9±1.3 cm, whereas it was 11.6±2.3 cm on those of the wild type (Fig. 6). These data indicate that the pathogen defense response was dramatically activated in *cea62* mutant plants.

### The reduced wounding response in *cea62* plants could be recovered by high levels of JA

**Figure 7:**
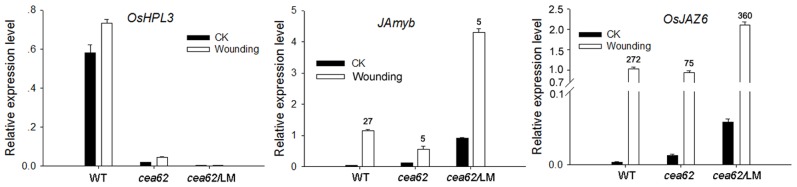
The wounding response of *cea62* plants. The expression of *OsJAmyb* and *OsJAZ6* were monitored in wound-treated wild type (WT) and *cea62* plants before and after the LM phenotype appeared. The results were obtained from three independent experiments.

Previous studies suggested that the expression of HPL genes was regulated by wound-induced transcriptional activation [30], [43]. HPL-derived oxoacid traumatin was identified as a wound hormone that triggers cell division at wound sites [38]. *OsHPL3* transcripts are known to be upregulated in response to wounding [41], suggesting that OsHPL3 plays an important role in wound signaling. We thus wounded the leaves of wild-type and *cea62* plants both before and after the LM phenotype appeared, and monitored the expression of wounding responsive genes. *OsJAmyb* is induced rapidly by JA or wounding and is involved in the JA-mediated signaling pathways in rice [44]. In the wild type, *OsJAmyb* and *OsJAZ6* transcripts were induced 27- and 272-fold, respectively, by the wounding treatment (Fig. 7). In contrast, *OsJAmyb* and *OsJAZ6* transcripts were induced only 5- and 75-fold, respectively, following wounding of *cea62* plants that did not yet exhibit the LM phenotype. This suggests that mutation of *OsHPL3* affects the wounding responses of *cea62* plants (Fig. 7). After the LM phenotype appeared in *cea62* plants, in which the concentration of JA was elevated, *OsJAmyb* and *OsJAZ6* transcripts were induced 5- and 360-fold by wounding treatment, respectively (Fig. 7). This suggests that the high levels of JA in wounded *cea62* plants recovered the induction of *OsJAZ6*, but did not recover the induction of *OsJAmyb*. These data imply that the AOS and HPL branches of the oxylipin pathway also undergo crosstalk in response to wounding. However, the details of this interaction are not clear.

## Discussion

Previous studies showed that both the AOS and HPL branches of the oxylipin pathway mediate plant responses to diverse biotic and abiotic stresses [1], [2] and suggested that regulatory crosstalk may exist between these branches of the pathway with respect to biosynthesis and signaling [39]. However, this possibility has not been supported by direct evidence. Here, we identified a natural HPL-depleted mutant in which JA production was dramatically increased and JA signaling was activated. Furthermore, activation of JA biosynthesis and signaling by OsHPL3 depletion varied at different developmental stages. The activation of JA signaling in turn elevated the defense responses and enhanced resistance to *Xoo* in the mutant plant. These findings suggest that these two branches of the oxylipin pathway cooperate with each other to contend with various environmental stresses and greatly advance our understanding of the relationship between these two branches of the oxylipin pathway. In addition, the *cea62* mutant identified here will provide valuable genetic tools to further dissect the complex interplay in oxylipin-mediated signaling networks.

**Figure 8:**
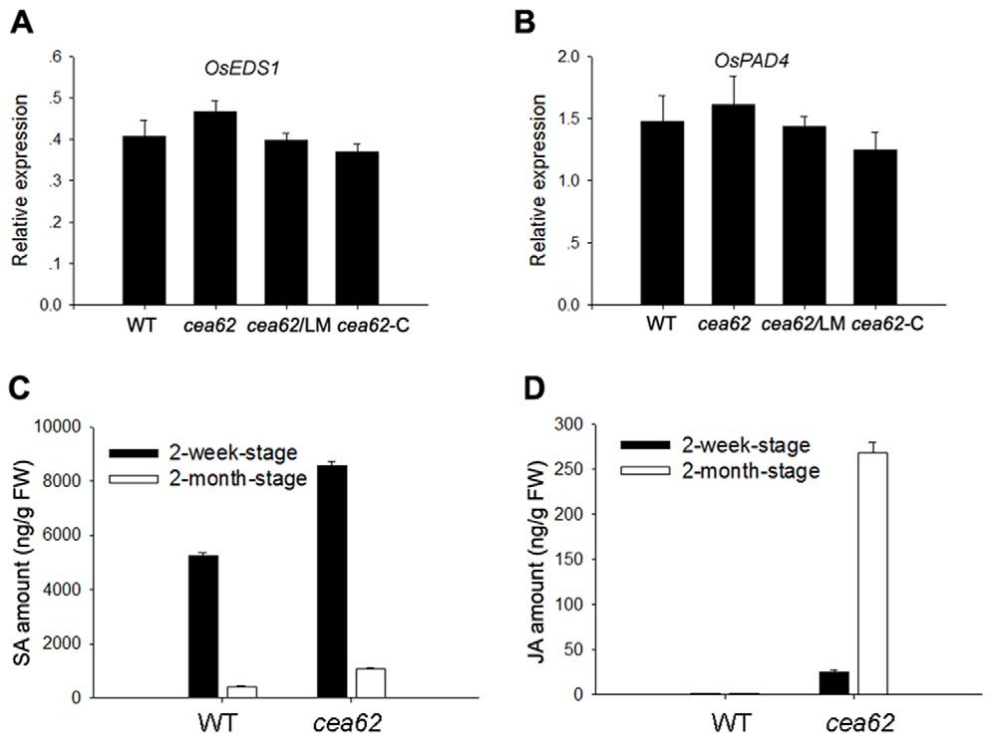
The expression of SA-regulated genes and quantity of SA and JA in the leaves of the WT and *cea62* mutant before and after the lesion mimic phenotype appeared. The leaves of 2-week-old and 2-month-old wild-type (WT) and *cea62* mutant plants were used to monitor the expression of the SA-regulated genes (A) *OsEDS1* and (B) *OsPAD4* and to quantify (C) SA and (D) JA levels. Each bar is the mean ± SD of three replicate samples.

We demonstrated that *OsNPR1* expression was induced in a development-dependent manner, as were JA-related genes, in *cea62* plants (Fig. 5). NPR1 was previously shown to function as a key regulator of salicylic acid (SA)-regulated systemic acquired resistance (SAR) in Arabidopsis, and transgenic rice plants overexpressing *OsNPR1* exhibited the lesion mimic phenotype and enhanced disease resistance to the *Xoo* pathogen [45], [46]. To verify that SA signaling is involved in generating the *cea62* phenotype, we monitored the expression of SA-related genes, such as *OsEDS1* and *OsPAD4* [46], in the *cea62* mutant. There was no difference in the expression of these genes during different developmental stages, in either the wild type or the mutant (Fig. 8A and B). We then detected the SA and JA levels in wild-type and *cea62* plants before and after the LM phenotype appeared. The SA level in *cea62* plants was found to be 1.6 times higher than that in the wild type at two weeks after sowing, and 2.5 times higher at two months after sowing (Fig. 8C). SA levels were lower at two months after sowing than at two weeks after sowing, in both the wild type and *cea62* mutant (Fig. 8C). In the *cea62* mutant, JA levels were 15 times greater than in the wild-type plant at two weeks after sowing and 162 times greater at two months after sowing (Fig. 8D). In contrast, there was no difference in JA production between these two developmental stages in the wild-type plant (Fig. 8D). Combined with the gene expression data, including the microarray data, these findings suggest that SA signaling may be involved in the defense response to *Xoo* in *cea62* plants, but that overproduction of JA and the activation of JA are mainly responsible for the mutant phenotype, including the enhanced pathogen disease resistance.

A series of studies revealed no obvious phenotypic differences between the HPL-depleted transgenic plants generated by anti-sense transformation and untransformed plants [23], [38], [39]. In Arabidopsis, it was demonstrated that *HPL*, which facilitated the production of C_6_ volatiles, was differentially expressed in the Columbia (Col) and Landsberg *erecta* (L*er*) ecotypes due to a 10-nt depletion polymorphism in the first exon of the gene in the Col ecotype. This depletion eliminated the ability of the Col ecotype to synthesize full-length HPL protein and the resulting products [42]. It was concluded that HPL signaling in the Col ecotype was dispensable for normal growth and development and that HPL was not replaced by an alternate enzyme capable of cleaving 13-HPOD [42] in Arabidopsis. In contrast, the normal growth and development of the *cea62* mutant was greatly affected by depletion of OsHPL3 and the *cea62* mutant displayed a severe phenotype, including cell death and lesion formation in leaves, semi-dwarfism and partial sterility (Fig. 1). Most significantly, JA biosynthesis, JA signaling and defense responses were continuously activated at specific developmental stages in the *cea62* mutant. A detailed inspection of differentially expressed gene profiles in *cea62* and wild-type plants by microarray analysis revealed that intense defense and stress responses were constantly initiated, even in the absence of pathogen infection and abiotic stress. Most differentially expressed genes functioning in these responses encode reactive oxygen species (ROS)-generating enzymes, heat shock proteins, JA biosynthesis and signaling proteins, glutathione S-transferases, pathogenesis- and disease resistance-related proteins, ethylene and gibberellin biosynthesis and responsive proteins, and abiotic stress-induced proteins, such as ABA-, salt-, light- and drought-induced proteins (Table 1). This clearly demonstrates that the HPL branch of the oxylipin pathway is indispensable for normal growth and development in rice. Furthermore, crosstalk between the AOS and HPL branches either involving substrate competition or signaling regulation plays an important role in the adaptation to environmental cues during plant growth and development. This communication helps establish a division-of-labor mechanism whereby the AOS and HPL branches of the oxylipin pathway perform distinct biological roles to provide effective protection against various invaders at a reduced metabolic cost. Actually, it is metabolically expensive for *cea62* plants in which the function of the OsHPL3 branch was eliminated to activate JA-governed defense responses in the absence of dangerous invaders. Interestingly, the *cea62* mutant displayed the LM phenotype three weeks after sowing in the greenhouse, but only two weeks after sowing in the field (data not shown). All these data suggest that the function of the HPL branch of the oxylipin pathway is to sense and protect the plant against various minor environmental stresses, such as light, temperature, ozone and wounding, and further to activate and cooperate with the JA-governed defense signaling pathway in response to severe stress by communicating with the AOS branch of the pathway.

Gomi [23] reported that *OsHPL2* is involved in white-backed planthopper-induced resistance to bacterial blight *Xoo* in rice. In addition, *OsHPL2*-overexpressing transgenic rice and (*E*)-2-hexenal-treated wild-type rice showed enhanced resistance to *Xoo*, which was proposed to be due to the negative effect of elevated levels of C_6_ volatiles and the upregulation of numerous defense-related genes on the growth of *Xoo* in rice [23]. Although the role of the JA signaling pathway in this interaction was not explored in that study, microarray analysis revealed that many JA-induced genes, such as *JAMyb*, *LOX2*, *AOS* and *PR1b*, were significantly induced by white-backed planthopper (WBPH) infestation [23]. The JA signaling pathway may cooperate with the HPL branch of the pathway to enhance resistance to *Xoo*. A recent report states that OsHPL3 functions in defense responses against different invaders [47]. The *hpl3-1* mutant was susceptible to the rice brown planthopper, but had enhanced resistance to the rice striped stem borer compared with the wild type [47]. Furthermore, *hpl3-1* plants showed increased resistance to bacterial blight *Xoo* [47]. The altered defense responses of *hpl3-1* plants to different invaders were largely ascribed to the elevated JA and SA levels or changed volatile niches [48]. Actually, the SA level was constitutively elevated in the *cea62* mutant, but the level of JA only increased at specific developmental stages, even in absence of an invader. Our microarray data showed that over a dozen P450 genes were differentially expressed in *cea62* plants, which provides clues as to the identity of the changed volatile niche for *hpl3-1* (Table 1). These data all suggest that crosstalk exists between the HPL and AOS branches of the oxylipin pathway in the plant defense response.

We have demonstrated that depletion of OsHPL3 greatly activates the JA-governed defense response in a development-dependent manner. This finding provides insight into the function of crosstalk between the HPL branch and AOS branch of the oxylipin pathway and suggests a novel strategy for manipulating rice disease resistance by modifying the communication between these two branches of the oxylipin pathway.

## Materials and Methods

### Plant materials and growth conditions

The rice (*Oryza sativa* L.) *cea62* mutant is in the Nipponbare (ssp japonica) background. Rice plants were cultivated in the experimental field of the Institute of Genetics and Developmental Biology (IGDB) in Beijing. An F2 mapping population was constructed by crossing *cea62* (Japonica) with Minghui 63 (Indica). Rice plants used for the treatment were grown from seed under glasshouse conditions [25°C, 60–80% relative humidity] to the five-leaf stage.

### Histochemistry

Because the leaves of the *cea62* mutant exhibit the lesion-mimic phenotype three weeks after sowing, leaf samples were harvested for histochemical analysis after lesions appeared (21 days and 30 days after sowing). ROS accumulation was detected as described previously [48], with some modifications. For DAB staining, which was used to detect H_2_O_2_, leaf samples were vacuum-infiltrated (three cycles of 5 min each) in 0.1% 3,3′-diaminobenzidine (DAB) containing 10 mM MES (pH 6.5), and the leaves were placed in the dark for 18 h at 28°C and cleared in boiling ethanol (95%) for 10 min. The cleared leaves were examined and photographed in 70% glycerol. Superoxide (O_2_^−^) accumulation was detected by staining with 0.5% nitro blue tetrazolium (NBT). The leaves were vacuum-infiltrated (three cycles of 5 min each) in 0.5% NBT in 10 mM potassium phosphate buffer (pH 7.8) for 16 h in the dark. Reactions were stopped by transfer to 90% ethanol at 70°C until the chlorophyll was completely removed, and the cleared leaves were examined and photographed in 70% glycerol. To detect dead cells, trypan blue staining was performed on fresh leaves, as described previously [48]. Briefly, samples were immersed in lactic acid-phenol-trypan blue solution (LPTB; 2.5% trypan blue, 25% (w/v) lactic acid, 23% water-saturated phenol and 25% glycerol in H_2_O) at 60°C, and vacuum infiltrated (three cycles of 5 min each). Leaf samples in LPTB were boiled in water for 2 min and then cooled to room temperature. Then, samples were destained for 3 days by replacing the LPTB solution with a chloral hydrate solution (25 g in 10 ml of H_2_O), and then equilibrated with 70% glycerol and mounted for microscopy.

### Map-based cloning of *CEA62*

For map-based cloning of *CEA62*, an F2 mapping population was constructed by crossing *cea62* with the Indica cultivar Minghui 63 (MH63). The *cea62* locus was first mapped between markers S2-290 and S2-827 of chromosome 2 using 368 F2 mutant plants. Another three molecular markers were developed and the underlying gene was further narrowed down to a 37-kb region using markers S2-570 and S2-632 and 1200 F2 plants. Nine predicted ORFs exist within this 37-kb interval: Os02g01960, Os02g01970, Os02g01980, Os02g01990, Os02g02000, Os02g02010, Os02g02020, Os02g02030, and Os02g02040 (Table S1). Sequencing analysis revealed that Os02g02000 contained a single base substitution.

### Complementation test of the *cea62* mutant

For complementation of *cea62*, the 6930-bp genomic DNA fragment containing *CEA62* (*OsHPL3*) driven by its native promoter was obtained by digestion of BAC AP004752 with EcoRI and SpeI and subsequently ligated into binary vector pCambia 1300 (Data S1). The plasmid was introduced into *Agrobacterium tumefaciens* AGL-1 by freeze-thaw transformation. Then, the mutant *cea62* callus was transformed by an Agrobacterium-mediated method as described previously [22].

### Quantitative RT-PCR analysis

Total RNAs were extracted from the leaves of rice plants using a TRIzol Kit according to the user's manual (Invitrogen). Three micrograms of total RNAs was treated with DNase I and used for cDNA synthesis with M-MLV Reverse Transcriptase (Promega). Real-time PCR experiments were performed using gene-specific primers (Table S3) and LightCycler® 480 SYBR Green I Master with the LightCycler® 480 Real-Time PCR System (Roche). Rice *Actin1* was used as an internal control.

### Enzyme assays

The hydroperoxide-metabolizing activity of *cea62* and the wild-type plant was detected by monitoring the absorbance decrease at 234 nm on The Synergy™ Mx spectrophotometer (BioTek Instruments). Enzyme assays were performed at room temperature in 1 mL of 50 mM sodium phosphate (pH 7.0) with 20 µl of crude extracts of wild-type, *cea62* or *cea62*-C plants. Crude extracts were obtained by homogenizing the plant leaves in enzyme buffer containing 1% Triton 20 (1 g leaf tissue per 10 ml buffer), and then spinning at 14000 g for 30 minutes. Reactions were initiated by adding 15 µl of 4 mM hydroperoxide substrate solution (13-HPOT or 13-HPOD; Cayman Chemical) to the reaction system. Two control reactions were performed simultaneously, one a blank control in which the plant crude extract was omitted, and the other a sample control that lacked substrate. These experiments were performed in triplicate for each enzyme activity experiment.

### Oligo DNA microarray analysis

The Affymetrix Rice Genome Array containing 51,279 transcripts, including 48,564 japonica transcripts and 1,260 indica transcripts, was used for the microarray analysis to monitor the differential expression between the wild type and *cea62* mutant. Total RNAs were extracted two months after sowing from the leaf blades of the *cea62* mutant that displayed the LM phenotype and from those of wild-type plants. Three replicates were performed for both the mutant and the wild type. All microarray procedures and data analyses were performed according to the manufacturer's instructions. Analysis was performed using an ANOVA-false discovery rate (ANOVA-FDR) p-value of <0.05. Spots with changes in expression were extracted based on the criterion of a twofold increase or decrease in expression. Functional classification of the differentially expressed genes was carried out using the tools for GO categories (http://plexdb.org and https://www.affymetrix.com) and revised manually.

### Quantification of JA, SA and C_6_-aldehydes

Leaf tissues (200 mg fresh weight) from wild-type and *cea62* plants were harvested and immediately frozen in liquid nitrogen. JA and SA was extracted and quantified by GC-MS as described previously [49]. C_6_-aldehyde volatiles in the leaf blades of rice were measured according to the method of Chehab et al. [32]. The aldehydes were quantified after careful preparation of calibration curves with n-hexanal and (*E*)-2-hexenal as standards.

### Bacterial inoculation

To evaluate bacterial blight disease resistance, plants were inoculated with *Xantho-monasoryzaepvoryzae* strain T1 at the seedling stage by the leaf-clipping method [50]. Disease was scored by measuring the lesion length at 3 weeks after inoculation. Thirty leaves, obtained from 30 plants (one leaf per plant), were inoculated, measured and subjected to statistical analysis. Three independent replicates were performed.

## Supporting Information

Data S1 The Genomic DNA sequence used to complement the *cea62* mutant. The genomic DNA sequence containing the OsHPL3 gene derived by its native promoter used for the complementary test was got from BAC AP004752 digested by EcoRI and SpeI.
(DOCX)

Figure S1 Fertility of the cea62 mutant. Pollen fertility of the wild type (A) and cea62 (B) was estimated by I2-KI solution staining. Fertile pollen stain blue. Scale bars, 50 µm. Seeds of the wild type (C) and cea62 mutant (D) plant. Scale bars, 1 cm.
(TIF)

Figure S2 Sequence of OsHPL3 and phylogenetic analysis of AOS and HPL proteins from Arabidopsis and rice. (A) The deduced amino acid sequences of OsHPL3. The star indicates the position of the mutated base in cea62, which forms a stop code. (B) Phylogenetic tree of reported HPL and AOS proteins in Arabidopsis and rice based on their full-length deduced amino acid sequences. Plant species and accession numbers are: AtHPL, AAC69871; AtAOS, CAA63266; OsHPL1, AK105964; OsHPL2, AK107161; OsHPL3, AY340220; OsAOS1, BAD08330; and OsAOS2, AAL17675.
(TIF)

Figure S3 Heat map of the microarray analysis of the cea62 plant. P-value<0.05; fold change>2.
(TIF)

Figure S4 Verification of the differentially expressed genes identified by microarray analysis using qPCR. A and B, JA-related genes. C, senescence-related genes. D and E, pathogenesis- and disease resistance-related genes. F, abiotic stress-related genes. G, GA-related genes. I, HSP genes. The same set of RNAs used for the microarray analysis was used to verify these genes by qPCR. The gene ID numbers and primers used to amplify these genes are provided in Table S3.
(TIF)

Table S1 The candidate genes between S2-570 and S2-632 in BAC AP004752. The gene locus, the start and end sites of the coding sequence and genes description for these candidate genes from gramene (http://www.gramene.org/) were listed.
(DOCX)

Table S2 The differentially expressed genes in the *cea62* mutant identified by microarray analysis. Gene expression profiles in leaf tissues of the *cea62* mutant and wild-type plant after the lesion mimic phenotype appeared two months after sowing were monitored using the Affymetrix Rice Genome Array, and 1686 differentially expressed transcripts (2-fold cutoff, p-values<0.05) were detected.
(XLSX)

Table S3 Gene ID numbers and primers used for quantitative PCR expression analysis. The genes ID, primer sequences and notes for those genes used in this research were listed.
(DOCX)
