# Supplementary material for: Activation of the Jasmonic Acid Pathway by Depletion of the Hydroperoxide Lyase OsHPL3 Reveals Crosstalk between the HPL and AOS Branches of the Oxylipin Pathway in Rice
Source: PLoS One. 2012 Nov 29;7(11):e50089. doi: 10.1371/journal.pone.0050089 (PMC3510209; doi:10.1371/journal.pone.0050089)
Supplement: Data S1 — The Genomic DNA sequence used to complement the cea62 mutant. The genomic DNA sequence containing the OsHPL3 gene derived by its native promoter used for the complementary test was got from BAC AP004752 digested by EcoRI and SpeI. (DOCX) [file pone.0050089.s001.docx]

Data S1: The Genomic DNA sequence used to complement the *cea62* mutant. The genomic DNA sequence containing the *OsHPL3* gene derived by its native promoter used for the complementary test was got from BAC AP004752 digested by EcoRI and SpeI.

ttcaattcctatttagagcaagtacaatagcagaatacaagtcaacaagtaaactataacaccatatgaaagagagaggagagagaaagcgtgctacagattggtagcgagctactacacggattccaaaagttatatgtgtataagaggtgagactatatattaataatatagtaagcaactattgtatgagttgactattaaattggttataaatgatttggatttagtagttagctatactattaaacttttgctcttataagtaactatttgatagaatgagctattagattgactatagatgatttggagccagtggttggctatactattaaacttcctctcaaccatttggagccggtggttagctatactattaaaacttgggagaatttgaaccatgccacacaaaattttgtaaaatttgtgatatgccaccctgacccacatgtcattgactcatgtgggtcctacatgtcattgagatacgggtggcatatctcaaattttgcaaatataggttggcatggttccaacaaacccaatccgtggtagatgatgtccggcaagcgagcttcctggccatctttattcccgagttttacatattttgattttggaaaaaaaaagggaaagagatgattgagtggatcaaaagccctaaaccaattttaaattttaataaataaagaaagatcagaggtgattgagtggataaaaaccctcaaccaatttaaaaagatcaagaatgatccaattttaaaacatctttgccgaatttgatgggaatgatggcatcggcatcggcatcttatggacagtatgtgcgagctgagccggcttctctcgtggactggccgtaaagagactgaaaatcgtggactgggccgtaaagagactgaaaaggaagaaaagattgggctttagtccaaaccgggactgaaaatgaagaatttattagctttcagtaaaaaaaatatttttcaatttatttctcacctcccaacaactatatattctatttctgaaaaaatatctttacttattacaacgactgacatttttcatagcattagagaaaaaaaaccatgcatgattatgtgttgcaaatgctccttactcccgtagcacggatgaatgatttacgggattcagttttatctaatttttctcattttatctgatttttgtatcgagttcttgtttatcgtaagaggatttagtatgaataatttcacattataataggtgtgaaataaattctctaaatcaccctgagattaactgaacaaaatcacccctggtgtgaaattaccctcaattattttttgtgtgagagattaaccgagcaaaatcaccccaatccttttgtgtgagagattaaccgaactaaaatcacccctcgtgtgatagattaaccgaacaatgcaaaatagaaaaatatccccaaccatatatactccctctatttcaaaatgtttgacaccgttgactttttaacacatgatcgttcgtcttattaaaaaaatttgtgaaatatgtaaaactatatgtatacatcaaagtatatttaacaataaatcaaatgatataaaaagaataaataattacttaaattttttgaataagacgaatggtcaaatatgtacttaaaaaatcaacggtgtcaaacattttgaaatggaaatggagggagtatggattaagaaagacacgttaaggatacaaggaaccctaacaaacattgtgtctcaaaacggcagcaaccaatcgtagacgcgatgatggcccacgtaatctgatttggttatacaaacgtgttgccacactgtggcagcctcctcctccgtgtacttgtgaacgctccagctaattttgtcatacacgcctagcacactagagtcagtgtcataacgcaagctaccacacgtagctgataagtccgatcgtcgccgcgcgccgcgccatggtgccgtcgttcccgcagccggccagtgcggcggcggcgacgcggccaataccggggagctacggcccgccgctgctcggcccgctccgcgaccgcctcgactacttctggttccagggccccgacgacttcttccgccgccgcgccgccgaccacaagagcaccgtgttccgcgccaacatcccgcccaccttccccttcttcctcggcgtcgacccgcgcgtcgtcgccgtcgttgatgccgccgccttcaccgcgctcttcgacccggccctcgtcgacaagcgcgacgtcctcatcggcccctacgtccccagcctcgccttcacccgcggcacccgcgtcggcgtctacctcgacacccaggaccccgaccacgcccgcaccaaggccttctccatcgacctcctccgccgcgccgcccgcaactgggccgccgagctccgcgccgccgtcgacgacatgctcgccgccgtcgaggaagacctcaacagggcccctgaccccgccgccgcctccgccagctacctcatcccgctccagaagtgcatcttccgcttcctctgcaaggcgctcgtcggcgccgacccggcggcggacggcctcgtcgaccgcttcggcgtgtacatcctcgacgtgtggctggcgttgcagctggtgccgacgcagaaggtgggcgtcatcccgcagccgctggaggagctcctgctccactccttcccgctgccgtcgttcgtcgtcaagcccgggtacgacctcctctaccgcttcgtggagaagcacggcgccgccgccgtgtccatcgctgagaaggagcacggcatcagcaaggaggaggccatcaacaacatcctcttcgtgctcggcttcaacgcgttcggcggcttctcggtgttcctgccgttcctggtcatggaggtcggcaagcccggccgggacgacctgcggcggcggctgcgggaggaggtgcgccgcgtgctgggcggcggcgacggcggcgaggccgggttcgcggcggtgagggagatggcgctggtgcggtcgacggtgtacgaggtgctccggatgcagccgccggtgccgctgcagttcgggcgggcgcggcgagacttcgtgctgcggtcgcacggcggcgcggcgtacgaggtgggcaagggcgagctgctgtgcgggtaccagccgctggccatgcgcgacccggcggtgttcgaccggccggaggagttcgtgccggagaggttcctcggcgacgacggcgaggcgctgctgcagtacgtgtactggtccaacgggccggagaccggcgagccgtcgccggggaacaaacagtgtgccgccaaggaggtggtcgtcgccaccgcgtgcatgctcgtcgccgagcttttccggcggtacgacgacttcgaatgcgacggcacctccttcaccaagctcgacaagcgggagctcactcccagctaagctttgctgccgccattctctcactcgatctccatgcacatatgcatgaagaaattaattaaattcaagttgctagctccattttttctctttgagctgctgataaaaaaaacatctctattcttctgtgcaataagccaataattaagcattaatcagagcgtacaagtaaaaattgttttcactgttttatgtggatatatatatgtacagggatccaccaaaattaatttgatactacgtagtacgacatttgtttagggatcaacgattcacgtttcacttttttttacagcgtcaacatatataaacacatagatatgagagtatttaatatacttattactgacatactcgcttcgtcccataaaaaaaaaccaaatcctatgtatgaatggagtattctagtagtacagtagtgtaaaaaaccgccaatagattgatcctgaatttcaggtcaaggagacaaagcaactactccatagaatcatagtattaggatatgtttcatccagtaaaaaaatatatttgaagatggggagtaagaaacacgaaacggaaaaacgagtgaagaagagaagacatttcagggtctgcttgtttctgatgccaaaagcatttttttagataatgccaaatgcgtggacagaatgcttgttctaataccaaatgcatggacagaatggttgataggtatctgcttgtatcttgctcgatggaaaccgcatggacagcgacgcatttcttctccttttgcatgttactactgaacggttgacacaggatatattactggcgaaaaagtacatgttaacatgggttcaaacagtaacacgcatttcaatcaagtgtgcaagtgcaaacacaaaatgatcgtctgtaaccgtagcgcgtaaacaacaagacgtacagttatactacctccattccaaaattttgacgccgttgacttttttaaaaatgtttgtccgttcgtcttattcaaaaaatttaagtaattgttaattctttttctatcatttgatttattgttaaatatacttttatgtatacgtatagttttacacattttacaaatttttttaaataagacgaacggttaaatatatttaaaaaaagttaacggcgtcaaacatttagggaaggagggagtatatgccaaacatatccctgtatggctatattgattggcaacaaatacagtactattgatcccgtgttgtgccaaccacaacagcgacaagtatcctgtcaatttgtggtgctaacaacttgccattgataagtccagattaactagaatagacgcaccaaggcacatacatgtaaatgctagctcctggataggcaaaattgatccaaagaccagctaaactgccaaatcgttgggaaagtttacataaatgtgtctcccttgcaggtaaacaaaaacatatagtgagctgaagaaggctagttcttcgtgcaagagatcagctgtgcaatcctcataaaagggatcggctgatcatagctgatgaagaaaatgaatttgttgatttcatgaataacagtggcactaaaaggcattctgaatataaatgaaaaatacagtgttagaaagacaccaaaaacaaacaaacaaagacttgcaaggaaaacagtacaagacaaacagtcaccatagttctacttctttcacatgtgaaacatggctaggaaaggaaaatgaaaacctagacatgtaggaattgagtatacgagaattgccatggtttgtaatttgtactctgtagtttgtaccataggcattgcatttattcatttactatggcaagagaagaacagagaaccaagagcagttcaagaatagatagcagtctgttagaggagcgagttcaatgcagaagagaagttataatcaattggtatggaaacaatggatgtatcaaatggcatcggaaaatgtcgttgctgcactcacatatctagctacaaaaaggacagatggcttgaaggaggagctagctacgtacatcatgatggtattattccaaatacaatctgaaacaggtttgtagcctgagtttaaatgataaaaaaaggagtaactgaatataattttaaatggttggtttgccaagtcagtaagctatacaagcacagcaatgtacaaggttatgaatatattaccaacaataatctgtctcaatgcagaaaacatgtcgaaataacaaagcacgaatgaacagagcaacgtctgtcatacaaaaaactaaaatttcttgtgtggtcttagcagagccacttatagccaacccaagtacagcgaaacgtagtcatggcagcttaatttctcaatgaaattcataacaagcacatgttcattgattattaattaaacatgttttcttttgctaaggcaggtaaatgtacctgcccatgtttgcctctatattggaattaatttgtttgttttctttccatataaaaaagtgctgggaagcttctattgcatttgtatacacttcttatatattctgtaaattagttaacttattattattattattatctcttacttatggaagaaaagtaattaaccaatgttgcgtgtcctcacaaggatgtgtttgttctagttctcgtaaaaagaaaagaaaagaaaaggaaaggaaagagaggaggtgggtttggcccaactagaaaggcatctcgtacgtaagcccacttagcccaactagagaacgttgtaggggttgcgggaatcaacagccgccgggagaggttaattagggttccaaacttgcatggagcgagagatcgagatgagcgagatggagacgggtaaggcgatcgagttggtgttgaagcggttggatgaggtgcgtacgcgcatggatggggtggagaagctgttgcgtatactcatctgcagggaggagaagcgagcgaaggttgggaggaggaggaggaggtcgacaagatgcagatggtgacggcggcggcaggtggcggtagcagcagcaggaggaggaaagccatcaagagaaactttaaggctaaagaagcagaccgcaaggagacggaagaggagggcaatcaggtgaagaagatcaccagccagcagctgtaagtgtatttaagtactccctccatattttaatgtaggatgccgttgactttttaacaccactcgtcttatttaaaaattatatgtaaatataaaaatacttatgtcaaaaattttatgcaaatataaaaatacttatgtcatgcttaaagaatatttgatgataaattaagtcgcaataaaataaattataattacataatttttttaataagacgaaaggttaaacgtttgtaaaaaaatcaacagcgtcatacattaaaatacggatacggatagagttttatctgtaccgatggatgatccgatccagctattatccatcatccattctgagtttattggtaatgatgatactactgatatggtcaatttaaccctaact
