# Supplementary figures and images for: Activation of the Jasmonic Acid Pathway by Depletion of the Hydroperoxide Lyase OsHPL3 Reveals Crosstalk between the HPL and AOS Branches of the Oxylipin Pathway in Rice
Source: PLoS One. 2012 Nov 29;7(11):e50089. doi: 10.1371/journal.pone.0050089 (PMC3510209; doi:10.1371/journal.pone.0050089)

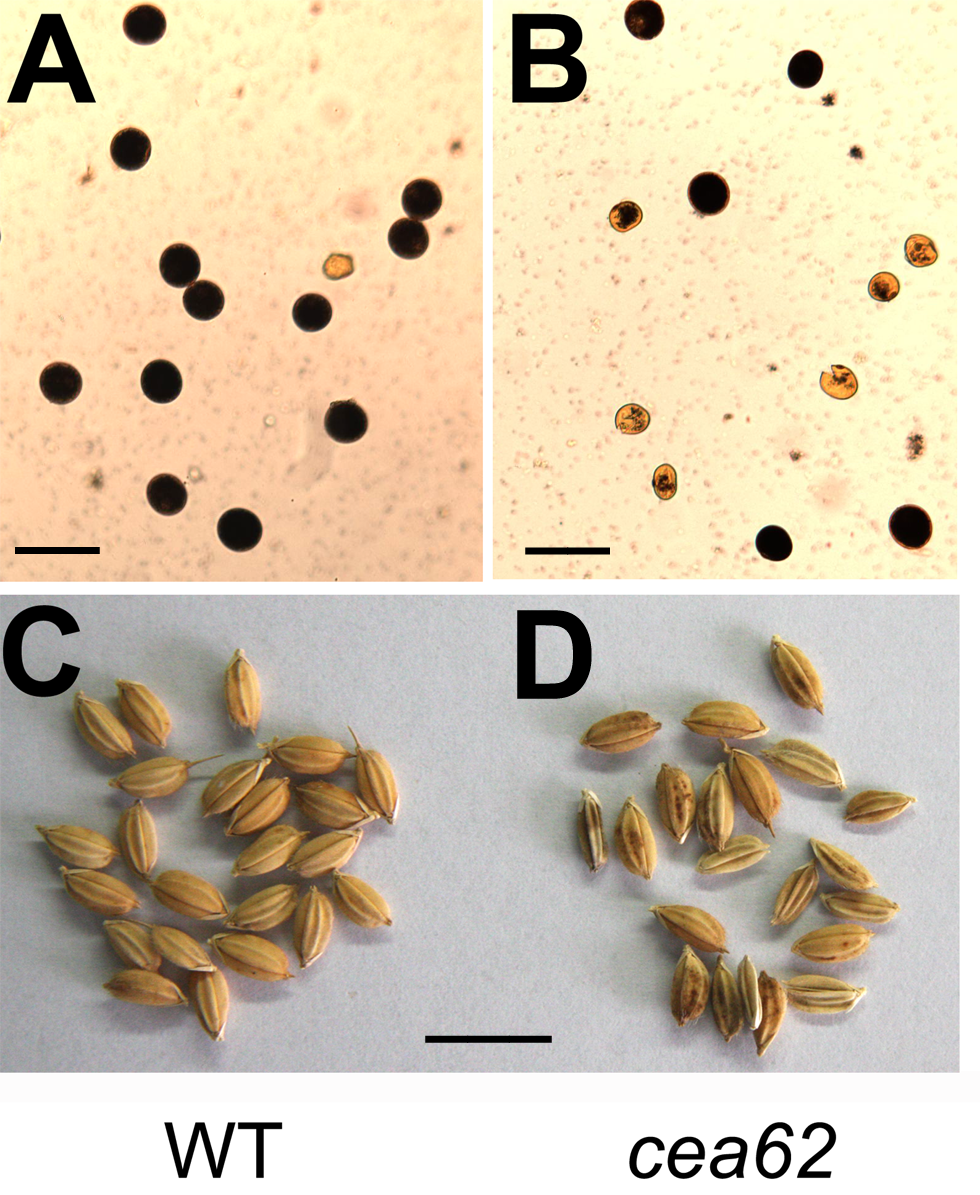

Supplement: Figure S1 — Fertility of the cea62 mutant. Pollen fertility of the wild type (A) and cea62 (B) was estimated by I2-KI solution staining. Fertile pollen stain blue. Scale bars, 50 µm. Seeds of the wild type (C) and cea62 mutant (D) plant. Scale bars, 1 cm. (TIF) [file pone.0050089.s002.tif]

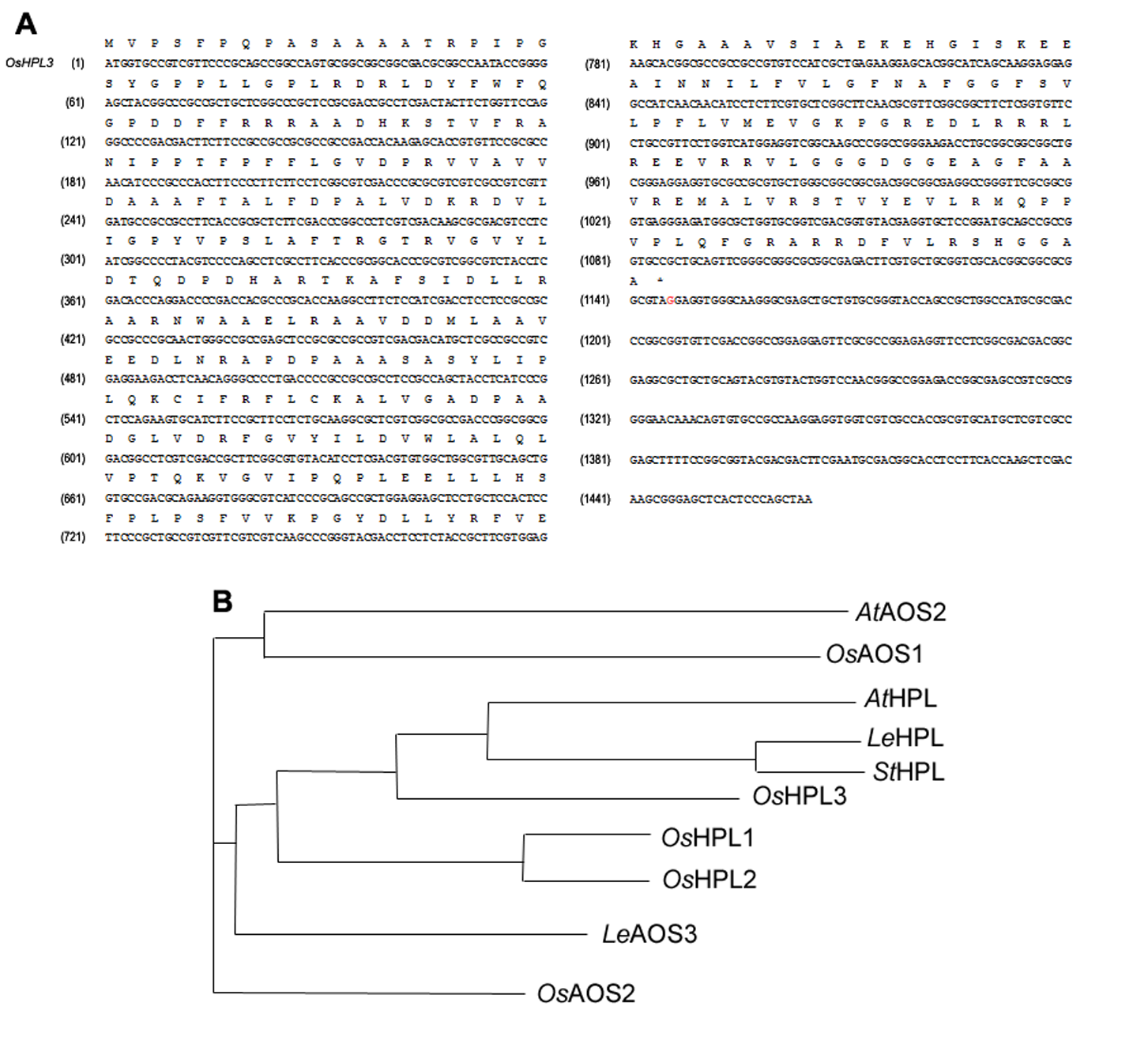

Supplement: Figure S2 — Sequence of OsHPL3 and phylogenetic analysis of AOS and HPL proteins from Arabidopsis and rice. (A) The deduced amino acid sequences of OsHPL3. The star indicates the position of the mutated base in cea62, which forms a stop code. (B) Phylogenetic tree of reported HPL and AOS proteins in Arabidopsis and rice based on their full-length deduced amino acid sequences. Plant species and accession numbers are: AtHPL, AAC69871; AtAOS, CAA63266; OsHPL1, AK105964; OsHPL2, AK107161; OsHPL3, AY340220; OsAOS1, BAD08330; and OsAOS2, AAL17675. (TIF) [file pone.0050089.s003.tif]

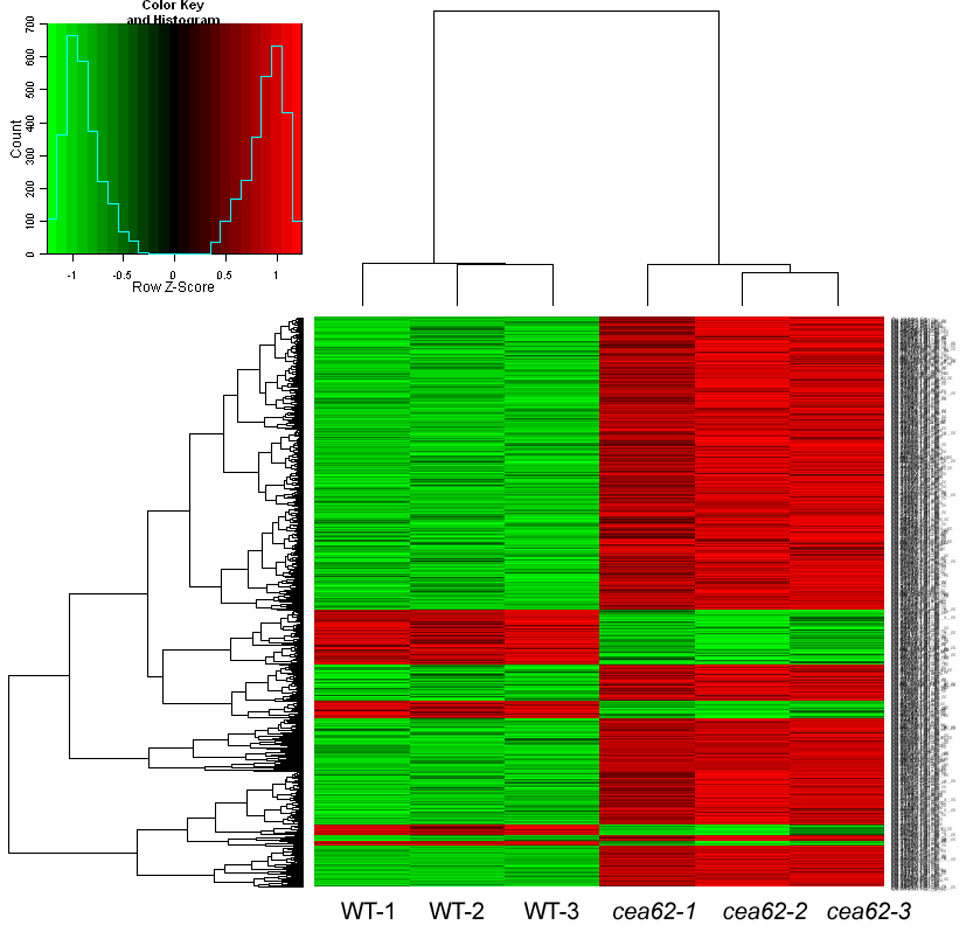

Supplement: Figure S3 — Heat map of the microarray analysis of the cea62 plant. P-value<0.05; fold change>2. (TIF) [file pone.0050089.s004.tif]

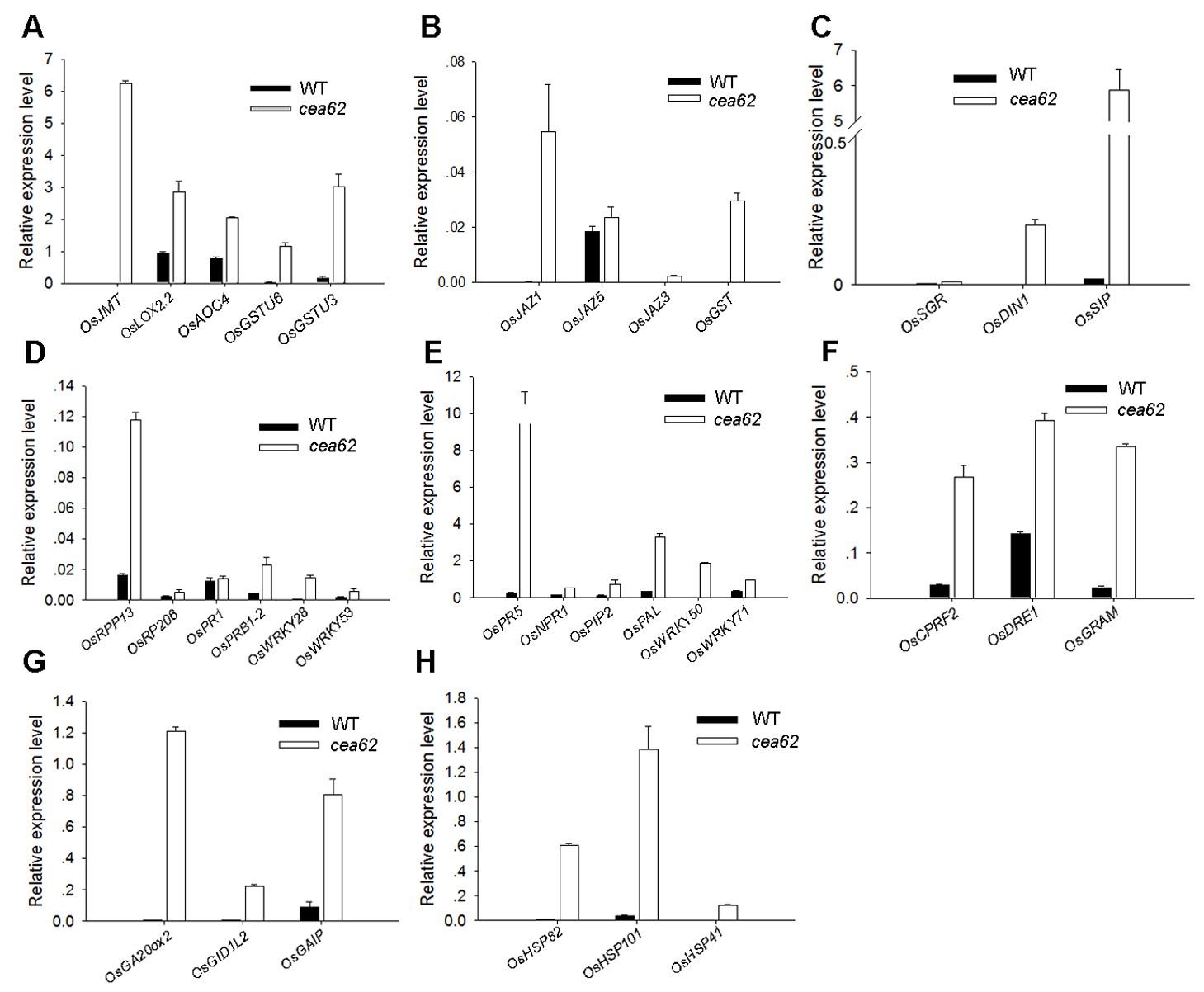

Supplement: Figure S4 — Verification of the differentially expressed genes identified by microarray analysis using qPCR. A and B, JA-related genes. C, senescence-related genes. D and E, pathogenesis- and disease resistance-related genes. F, abiotic stress-related genes. G, GA-related genes. I, HSP genes. The same set of RNAs used for the microarray analysis was used to verify these genes by qPCR. The gene ID numbers and primers used to amplify these genes are provided in Table S3. (TIF) [file pone.0050089.s005.tif]
