# Supplementary material for: Activation of the Jasmonic Acid Pathway by Depletion of the Hydroperoxide Lyase OsHPL3 Reveals Crosstalk between the HPL and AOS Branches of the Oxylipin Pathway in Rice
Source: PLoS One. 2012 Nov 29;7(11):e50089. doi: 10.1371/journal.pone.0050089 (PMC3510209; doi:10.1371/journal.pone.0050089)
Supplement: Table S1 — The candidate genes between S2-570 and S2-632 in BAC AP004752. The gene locus, the start and end sites of the coding sequence and genes description for these candidate genes from gramene (http://www.gramene.org/) were listed. (DOCX) [file pone.0050089.s006.docx]

Table S1. The candidate genes between S2-570 and S2-632 in BAC AP004752. The gene locus, the start and end sites of the coding sequence and genes description for these candidate genes from gramene (http://www.gramene.org/) were listed.

| **gene locus** | **start** | **end** | **gene description** |
| --- | --- | --- | --- |
| LOC_Os02g01970 | 996 | 5808 | expressed protein |
| LOC_Os02g01980 | 6585 | 9158 | anther-specific proline-rich protein APG precursor |
| LOC_Os02g01990 | 10510 | 12067 | CCT motif family protein |
| **LOC_Os02g02000** | **15464** | **17205** | **cytochrome P450 74A4, putative, expressed** |
| LOC_Os02g02010 | 19800 | 20850 | conserved hypothetical protein |
| LOC_Os02g02020 | 22708 | 25372 | expressed protein |
| LOC_Os02g02030 | 25601 | 26838 | expressed protein |
| LOC_Os02g02040 | 26933 | 33628 | protein kinase, putative, expressed |
