# Supplementary material for: Activation of the Jasmonic Acid Pathway by Depletion of the Hydroperoxide Lyase OsHPL3 Reveals Crosstalk between the HPL and AOS Branches of the Oxylipin Pathway in Rice
Source: PLoS One. 2012 Nov 29;7(11):e50089. doi: 10.1371/journal.pone.0050089 (PMC3510209; doi:10.1371/journal.pone.0050089)
Supplement: Table S3 — Gene ID numbers and primers used for quantitative PCR expression analysis. The genes ID, primer sequences and notes for those genes used in this research were listed. (DOCX) [file pone.0050089.s008.docx]

| Table S3: Genes ID and Primers used for quantitative PCR expression analysis. The genes ID, primer sequences and notes for those genes used in this research were listed. | | | |
| --- | --- | --- | --- |
| gene | Gene ID | Primer sequence | note |
| *OsEDS1:* | LOC_Os09g22450 | Forward: cattccaagaacgaggacactg  Reverse: caagactcaaggctagaaccga |  |
| *OsHSP82* | LOC_Os04g01740.1 | Forward: gggacctcgtcctcctcctctt  Reverse: GGCGTCGTCGTCGTCGATGTT | heat shock protein 82 |
| *OsHSP101* | LOC_Os05g44340.1 | Forward: CCCCTGTCCCACGAGCAACT  Reverse: CTGATCGGCCTCGCGCCATA | heat shock protein 101 |
| *OsHSP 41* | LOC_Os03g53340.2 | Forward: CAGCCTCGGCCTGGAGGAGAA  Reverse: GCACGAGCACGTCCACGTCAT | heat shock protein 41 |
| *OsGA20ox2* | LOC_Os01g61610.1 | Forward: GGAGCACCGCGTGATCGTGAA  Reverse: CCTCAAGCTGTGCCTTGCCCTT | gibberellin 20 oxidase 2 |
| *OsGID1L2* | LOC_Os03g57640.1 | Forward: GCAGGTGGAGATGGTGGAGTT  Reverse: CGTCACCGCCGTTGCCGTA | gibberellin receptor GID1L2 |
| *OsGAIP* | LOC_Os03g55290.1 | Forward: CAGGCACGACGACTGCCTCAA  Reverse: GGCCTCTTGCGAGCGCCAT | Gibberellin stimulated transcript related protein 1. |
| *OsGST* | LOC_Os09g20220.1 | Forward: GTGGCGTGGTTCCACGCCTA  Reverse: GGCCTTGAGGTAGAGCGCGAA | glutathione S-transferase |
| *OsGSTU6* | LOC_Os10g38340.1 | Forward: GAGCGAGTGCTCCAAGGGGAA  Reverse: GGCGGCGTCGAACTGGTTCA | glutathione S-transferase GSTU6 |
| *OsGSTU3* | LOC_Os10g38489.1 | Forward: GGTTTGTGCGCGTCGGTGAA  Reverse: GCTGCCCACTCTGCCTGCTT | glutathione S-transferase OsGSTU3 |
| *OsWRKY50* | LOC_Os11g02540.1 | Forward: GCGGTCAGCACGGCGCCTA  Reverse: CGATGTGCATCGGCGACCTGA | OsWRKY50 transcription factor |
| *OsWRKY28* | LOC_Os06g44010.1 | Forward: CGCAGCGCCGACGACAAC  Reverse: GCGGCTGGTGCTGTGAGTGCTT | OsWRKY28 transcription factor |
| *OsWRKY53* | AB190436 | Forward: CCACCTACGAGGGCAAGCAC  Reverse: GGGGAGCATCTCGAGGGTGTA | OsWRKY53 transcription factor |
| *OsWRKY71* | AB190817 | Forward: GCCGCACCGTCAGCACGA  Reverse: GACGAGCGCCGCCTTGAAGC | OsWRKY71 transcription factor |
| *OsBIERF3* | LOC_Os02g43790.1 | Forward: GCTCCGCATCGGCTCCGAGA  Reverse: CGGTGACCCGGAGGAGGAAGA | BTH-induced ERF transcriptional factor 3 |
| *OsERF1* | LOC_Os01g21120.1 | Forward: CTGCTCCTCCGACGAGGTCAA  Reverse: GCGACGGCAGCTCGTAGTCTT | ethylene-responsive factor-like protein 1 |
| *OsERF15* | LOC_Os07g22730.1 | Forward: GTGGCTCGGCACGTTCGA  Reverse: GCTTGGAGTGCCGCCCCTT | ethylene-responsive transcription factor 15 |
| *OsERF2* | LOC_Os01g54890.1 | Forward: GGCTGTGGCTCGGCACCTT  Reverse: GTCGCTCATGCCGACCTGC | ethylene-responsive transcription factor 2 |
| *OsEIL3* | LOC_Os08g39830.1 | Forward: ggagacaggggtgttggctgat  Reverse: CCACTGTCGAAAGGATCTCCCAT | ethylene-insensitive3-like protein |
| *OsJAmeTase* | LOC_Os02g48770.1 | Forward: GCCATCGGTGGACGAAGTGAA  Reverse: CGTCACTGTCGTCCTGTGGAT | jasmonate O-methyltransferase |
| *OsLOX2.2* | LOC_Os12g37320.1 | Forward: GGGCGAGGAGGAGGAGTTCAA  Reverse: GGGTTCATCCCTGCCAGCGTT | lipoxygenase 2.2 |
| *OsAOC4* | OSJNBA0054H04.12 | Forward: GCAGGGCCCGTACCTGACCTA  Reverse: GGGGATGCCCTTGAGGTAGAA | allene oxide cyclase 4 |
| *OsJAZ6* | LOC_Os03g08310.1 | Forward: GGACATGCCGATCGCGAGGAA  Reverse: GCGCGAGTGCATGTGTCCAA | ZIM motif family protein OsJAZ6 |
| *OsJAZ13* | LOC_Os10g25230.1 | Forward: GGGTGCTGGTGCTCGACGAGT  Reverse: CGCTGCAGCGACGCCTTCCT | ZIM motif family protein OsJAZ13 |
| *OsJAZ3* | LOC_Os03g08330.1 | Forward: GAAGGCGTCGCTGCAGCGGTT  Reverse: CAGCGCGATGGTGAGGCTGTC | ZIM motif family protein |
| *terpene synthesis* | LOC_Os02g02930.1 | Forward: GGGATGACCTGGGCAGTGCCAA  Reverse: GCGGAGAAGCACTCCCGGTT | terpene synthase 2 |
| *OsDIN1* | LOC_Os06g50930.1 | Forward: CAGCAGGCCGGACACCGCTA  Reverse: GCTGCCATGAGGGATCTCTT | senescence-associated protein DIN1, |
| *OsSGR* | LOC_Os09g36200.1 | Forward: CGGCGACGGCAACCTGTTCA  Reverse: GTCTTCCTCGGGCGGCGCT | senescence-inducible chloroplast stay-green protein |
| *OsPOD2* | LOC_Os04g55740.1 | Forward: CACCCTCGCCACCTCGCTGAA  Reverse: CGGCCATGTCGGTGGCGTA | peroxidase 2 precursor |
| *OsPAL* | LOC_Os02g41670.1 | Forward: CGACCCGTGCAGCGCCAACTA  Reverse: GCGACCTTGGCGAACACCGA | phenylalanine ammonia-lyase |
| *OsPIP2* | LOC_Os02g47650.1 | Forward: CCACCTCGTCCACGCCGTCT  Reverse: GCCGCTCTGCCTCTCGGCAA | pathogen induced protein 2-4 |
| *OsPR5* | LOC_Os12g38170.1 | Forward: CGGTGCCCGCTGACGAGGA  Reverse: GACGTCGACGGTGCGGATGA | pathogenesis-related protein 5 |
| *OsPR1* | LOC_Os03g18850.1 | Forward: CTGGTGGAGGGCGGCGGCAT  Reverse: CCGGCTCGCCGGCGACCAT | pathogenesis-related protein 1 |
| *OsPRB1-2* | LOC_Os07g03600.1 | Forward: GGACCTGGGCTACGGCGAGAA  Reverse: CCCCTCGTGCACGTGTTGCT | pathogenesis-related protein PRB1-2 |
| *OsRPP13* | LOC_Os03g40194.1 | Forward: GAGGCTTCCTCCTGTGCAGT  Reverse: GCTGACCTCAATGTTGTCCATC | disease resistance RPP13-like protein 1 |
| *OsRP206* | LOC_Os12g07580.1 | Forward: CGTCTTCCAGTCCGGCAGGTA  Reverse: GTGGCGTCGCCGGTCTTCA | disease resistance response protein 206 |
| *OsNPR1-1* | LOC_Os03g46440.1 | Forward: GGACACAGGATGGACGTGAT  Reverse: GTAAGACGCCAATGCACAAGTA | regulatory protein NPR1 |
| *OsSIP* | LOC_Os01g24710.2 | Forward: CCCATGGCCCAGTCTATGAT  Reverse: GGCCAGAGTCTTGCAGTGGAA | salt-induced protein |
| *OsCPRF2* | LOC_Os08g26880.1 | Forward: GGCTATCAGCGCTCCGTGAGA  Reverse: GCATCCCTGAGCCCCATGC | light-inducible protein CPRF-2 |
| *OsGRAM* | LOC_Os12g29400.1 | Forward: GTGCACGTCGTGACGGTGGA  Reverse: CGCCACGTTGCTGGGAGGA | ABA-responsive protein, GRAM domain protein |
| *OsDRE1* | LOC_Os01g07120.2 | Forward: GTAGTCCCTGAGGTGCAGGAT  Reverse: CATCTCCCTCTTGGTACTCTT | DRE binding protein 2 |
| *OsHPL3* | LOC_Os02g02000 | Forward: CGAGGCGCTGCTGCAGTACGTGTA  Reverse: CTCCCGCTTGTCGAGCTTGGTGAA | hydroperoxide lyase 3 |
| *OsJAmyb* | Os11g45740 | Forward: ccgagcatggtgactagctcatctt  Reverse: ccttgcacccaaccgttaagctgtt |  |
| *OsPR1b* | B1096D0 | Forward: GTGTGCGGGCACTACACG  Reverse: CGGCTTATAGTTGCATGTGA |  |
| *OsPR10* | D38170 | Forward: CACCATCTACACCATGAAGC  Reverse: AGCACATCCGACTTTAGGAC |  |
| *OsLOX* | D14000 | Forward: GATGGCGGTGCTCGACGTGCT  Reverse: GCACCTGTTCTTGAGCTTTCTAT |  |
| *OsAOS2* | LOC_Os03g12500 | Forward: ctcgtcggaaggctgttgct  Reverse: acgattgacggcggaggtt |  |
| *OsACT1* | LOC_Os03g50885 | Forward: cagcacattccagcagat  Reverse: ggcttagcattcttgggt |  |
| *OsPAD4* | CX118864 | Forward: ccaacatgtaccgcatcaag  Reverse: ggttgtttcggtggtagtgg |  |
| *OsJAZ8* | LOC_Os09g26780 | Forward: CCAAACACGGCGGAAACAG  Reverse: GGTGGACGGGAAGTTCTCAAAG |  |
| *OsNPR1* | LOC_Os01g09800 | Forward: GGCAGGTGAGAGTCTACGAGGAA  Reverse: gctgtcatccgagctaagtgtt |  |
